# Highly Potent 1*H*-1,2,3-Triazole-Tethered Isatin-Metronidazole Conjugates Against Anaerobic Foodborne, Waterborne, and Sexually-Transmitted Protozoal Parasites

**DOI:** 10.3389/fcimb.2018.00380

**Published:** 2018-10-30

**Authors:** Sumit Kumar, Trpta Bains, Ashley Sae Won Kim, Christina Tam, Jong Kim, Luisa W. Cheng, Kirkwood M. Land, Anjan Debnath, Vipan Kumar

**Affiliations:** ^1^Department of Chemistry, Guru Nanak Dev University, Amritsar, India; ^2^Center for Discovery and Innovation in Parasitic Diseases, Skaggs School of Pharmacy and Pharmaceutical Sciences, University of California, San Diego, La Jolla, CA, United States; ^3^Department of Biological Sciences, University of the Pacific, Stockton, CA, United States; ^4^Foodborne Toxin Detection and Prevention Research Unit, Agricultural Research Service, United States Department of Agriculture, Albany, CA, United States

**Keywords:** *Entamoeba histolytica*, *Trichomonas vaginalis*, *Tritrichomonas foetus*, *Giardia lamblia*, metronidazole, cytotoxicity, isatin-metronidazole conjugates

## Abstract

Parasitic infections like amebiasis, trichomoniasis, and giardiasis are major health threats in tropical and subtropical regions of the world. Metronidazole (MTZ) is the current drug of choice for amebiasis, giardiasis, and trichomoniasis but it has several adverse effects and potential resistance is a concern. In order to develop alternative antimicrobials, a library of 1*H*-1,2,3-triazole-tethered metronidazole-isatin conjugates was synthesized using Huisgen's azide-alkyne cycloaddition reaction and evaluated for their amebicidal, anti-trichomonal, and anti-giardial potential. Most of the synthesized conjugates exhibited activities against *Trichomonas vaginalis, Tritrichomonas foetus, Entamoeba histolytica*, and *Giardia lamblia*. While activities against *T. vaginalis* and *T. foetus* were comparable to that of the standard drug MTZ, better activities were observed against *E. histolytica* and *G. lamblia*. Conjugates **9d** and **10a** were found to be 2–3-folds more potent than MTZ against *E. histolytica* and 8–16-folds more potent than MTZ against *G. lamblia*. Further analysis of these compounds on fungi and bacteria did not show inhibitory activity, demonstrating their specific anti-protozoal properties.

## Introduction

Anaerobic protozoan parasites *Trichomonas vaginalis* and *Tritrichomonas foetus* are the major causes of reproductive tract infections *viz*. trichomoniasis and bovine trichomoniasis (Kumar et al., 2015; Ravaee et al., 2015). Though men remain asymptomatic, trichomoniasis leads to urogenital infection in women via sexual transmission, resulting infertility, urethritis, vaginitis, preterm delivery and low birth weight, Bovine trichomoniasis, acquired by direct sexual intercourse, is asymptomatic in nature but earlier death of developing foetus was observed in some cases. Multilocus genotyping confirmed that the isolates obtained from cattle and pig represented the “bovine genotype” of *T. foetus* and the cat isolates represented a closely related “feline genotype” of *T. foetus* (Fang et al., 2015). The prevalence of *T. vaginalis* infection in the United States is estimated to be 2.3 million among women of 14–49 years and this increases with age (Sutton et al., 2007; Conrad et al., 2013). Moreover, women without any past history of sexual intercourse can still be affected with trichomoniasis (Kumar et al., 2012). Major health threats associated with trichomoniasis include transmission of HIV-1 (Fichorova, 2009), benign prostatic hyperplasia (Mitteregger et al., 2012), prostate cancer and pelvic inflammatory disease (Stark et al., 2009).

Two other anaerobic intestinal protozoans, *Giardia lamblia* and *Entamoeba histolytica* cause the gastrointestinal diarrheal diseases (Halliez and Buret, 2013), giardiasis and amebiasis. Acute gastroenteritis is considered as one of the leading causes of illnesses and deaths in children under the age of 5 years (Heresi et al., 2000; Youssef et al., 2000). Infection occurs through the ingestion of cysts in contaminated water or food and direct person-to-person contact. Upon ingestion of *G. lamblia* or *E. histolytica* cysts, trophozoitese merge from the cysts and multiply in the lumen of the small intestine, where *G. lamblia* attach to the intestinal mucosa (Navaneethan and Giannella, 2008). *E. histolytica* trophozoites invade the colon and causes amoebic colitis. While 50% of *G. lamblia* infection is asymptomatic, major symptoms of amebiasis and giardiasis include weight loss, loss of appetite, watery or bloody diarrhea, dehydration, bloating and abdominal cramps, cognitive impairment in children, and chronic fatigue in adults (Berkman et al., 2002; Hanevik et al., 2007).

**Figure 1:**
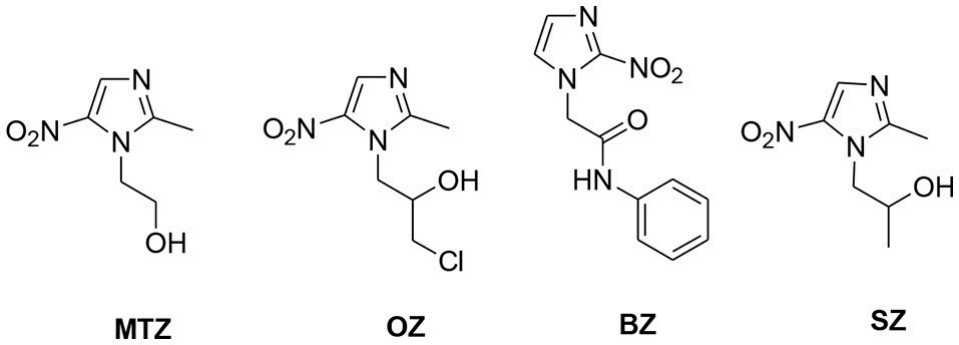
Structures of different derivatives of nitro-imidazoles.

Nitro-imidazoles such as ornidazole (OZ), benznidazole (BZ), and secnidazole (SZ) (Figure 1) are the widely used medicament to treat anaerobic infections but have lower efficacies than MTZ (Nash, 2002). MTZ, an effective synthetic drug introduced in 1960, showed strong inhibitory efficacies against Gram-negative anaerobic bacteria like *Helicobacter pylori* and protozoans such as *G. lamblia* and *E. histolytica*. It is the only therapeutic drug available till date against trichomoniasis (Cudmore et al., 2004; Sutherland et al., 2010). These outstanding achievements have encouraged researchers to focus on the development of nitro-imidazoles with imminent medicinal application. However, MTZ resistance has been observed in *E. histolytica, G. lamblia*, and *T. vaginalis* and thus the development of novel, non-cytotoxic and efficient scaffolds against amebiasis, giardiasis, trichomoniasis, and bacterial infections is desirable (Meri et al., 2000; Upcroft et al., 2005; Debnath et al., 2013).

**Figure 2:**
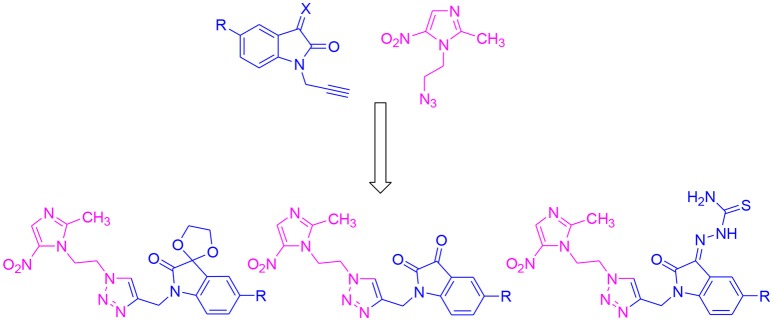
General Structure of target hybrid compounds.

Isatin is one of the important pharmacophores with wide application in drug discovery and it is the core constituent of many alkaloids, dyes, pesticides, and analytical reagents. Various studies from the literature show the anti-bacterial (Sarangapani and Reddy, 1994), anti-fungal (Pandeya et al., 1999), anti-inflammatory (Bhattacharya and Chakrabarti, 1998) and anticonvulsant (Popp et al., 1980) properties of isatin-schiff bases. Thiosemicarbazones are the class of Schiff bases that exhibit anti-parastic and anti-bacterial properties (Chellan et al., 2012). Moreover, the spiro compounds of isatin are also known to exhibit versatile biological properties. Earlier results based on 1*H*-1,2,3-triazole and β-amino-alcohol tethered isatin-β-lactam conjugates showed efficient inhibitory activities against *T. vaginalis* (Nisha et al., 2013). Results obtained with *N*-propargylated-isatin-Mannich adducts and *N*-propargylated isatin-quinoline Mannich adducts against *T. foetus* have also prompted the use of isatin as a pharmacophore (Nisha et al., 2014). Based on the anticancer (Vine et al., 2007; Kumar et al., 2018; Singh et al., 2018), anticonvulsant (Verma et al., 2004), antidepressant (Singh et al., 1997), anti-HIV (Bal et al., 2005), and anti-bacterial (Pandeya et al., 2000) properties of isatin, the present study explicates the synthesis and biological evaluation of 1*H*-1,2,3-triazole-tethered isatin-metronidazole conjugates as shown in Figure 2. Substitutions at C-5 position of isatin-ring as well as the introduction of thiosemicarbazide and ketocarbonyl at C-3 carbonyl of isatin have been carried out to ascertain the structure-activity relationship of the synthesized conjugates.

## Experimental section

### General information

Melting points were determined with open capillaries using a Veego Precision Digital Melting Point apparatus (MP-D) and are uncorrected. ^1^H and ^13^C NMR were recorded on JEOL 400, Bruker 500 MHz spectrometer, respectively using CDCl_3_ as solvent. Chemical shifts were reported in parts per million (ppm) using tetramethylsilane (TMS) as an internal standard and coupling constants *J* indicated in Hertz. Splitting patterns were indicated as s: singlet, d: doublet, t: triplet, m: multiplet, dd: double doublet, ddd: doublet of a doublet of a doublet, and br: broad peak. Mass spectrometric analysis was carried out on BrukermicrOTOF QII equipment using ESI as the source. Column chromatography was performed on a silica gel (60–120 mesh) using an ethyl acetate: hexane mixture as eluent (Kumar et al., 2017).

### General procedure for the synthesis of C-5 substituted isatin-spiro-ketals (2)

A solution of isatin **1** (1 mmol) in dry DMF was added drop-wise to a stirred suspension of NaH (1.2 mmol) in dry DMF at 0°C. The solution was then stirred for 15 min. followed by the drop-wise addition of 2-bromoethanol (1.2 mmol). The reaction mixture was allowed to stir at room temperature for 20 min. with subsequent heating at 80°C for 4 h. The progress was monitored by TLC. Upon completion, the reaction mixture was extracted with ethyl acetate (2 × 25 mL), washed with brine water (2 × 20 mL) and the combined organic layers were dried over anhydrous Na_2_SO_4_. The organic layer was concentrated under reduced pressure to afford the desired precursors.

### General procedure for the synthesis of *N*-propargylated C-5 substituted isatin (3)/isatin spiroketal (4)

To a stirred suspension of NaH (1 mmol) in dry DMF was added drop-wise a solution of C-5 substituted isatin (1 mmol) **3** /isatin-spiroketal **4** in dry DMF at 0°C. The mixture was stirred for 15 min followed by the drop-wise addition of propargyl bromide (1.2 mmol). The reaction mixture was allowed to stir at room temperature for 10 min. with subsequent heating at 60°C for 4 h with progress being monitored by TLC. Upon completion, the reaction mixture was extracted with ethyl acetate (2 × 25 mL) and washed with brine water (2 × 20 mL). The combined organic layers were dried over anhydrous Na_2_SO_4_, concentrated under vacuum to afford the desired precursors.

### General procedure for the synthesis of conjugates, 8a–e and 9a–e

CuSO_4_.5H_2_O (0.055 mmol) and sodium ascorbate (0.143 mmol) were added to a well stirred solution of 5-substituted 1-(prop-2-yn-1-yl)indoline-2,3-dione **3** (1 mmol) or 5-substituted 1-(prop-2-yn-1-yl)spiro[indoline-3,2^/^-[1,3]dioxolan]-2-one **4** and 1-(2-azidoethyl)-2-methyl-5-nitro-1*H*-imidazole **7** (1 mmol) in an ethanol:water (85: 15) mixture. The reaction mixture was allowed to stir at room temperature for 6–7 h and the progress was monitored by TLC. Upon completion, the reaction mixture was extracted with ethyl acetate and water and the combined organic layers were dried over anhydrous Na_2_SO_4_ and concentrated under vacuum to afford the desired conjugates which were purified via column chromatography using an ethyl acetate: hexane (70:30) mixture.

### General procedure for the synthesis of conjugate, 10a–e

Thiosemicarbazide (1 mmol) and glacial acetic acid in catalytic amount were added to a stirred solution of 5-substituted1-((1-(2-(2-methyl-5-nitro-1*H*-imidazol-1-yl)ethyl)-1*H*-1,2,3-triazol-4-yl)methyl)indoline-2,3-dione (**8**) in ethanol. The reaction mixture was heated to reflux for 2 h and the progress was monitored using TLC. On completion, yellow colored solid resulted and this was re-crystallized using an absolute ethanol to afford the desired conjugates.

## Materials and methods

### Biological evaluation

#### *In vitro* susceptibility assay against the bovine trichomonad *T. foetus* strain D1 and the feline trichomonad *T. foetus*-like strain C1

To perform the initial susceptibility screens on *T. foetus* D1 (from Lynette Corbeil, University of California at San Diego, School of Medicine, San Diego, CA, USA) and *T. foetus*-like C1 (from Stanley Marks, University of California at Davis, School of Veterinary Medicine, Davis, CA, USA), compounds were dissolved in 100% DMSO to obtain concentrations of 100 mM. Stock solutions were kept at −20°C. 5 microliter aliquots of these solutions were diluted in 5 mL of TYM Diamond's media (Hardy Diagnostics, Santa Maria, CA, USA) to obtain a final concentration of 100 μM. Metronidazole-sensitive D1 and C1 strains were cultured under anaerobic conditions. After 24 h, cells were counted using a hemacytometer. The IC_50_ value for the series of compounds was determined by inoculating a constant number of parasite cells in TYM medium and running assays of increasing drug concentrations, 0.02–100 μM, and performing a regression analysis on percentage growth inhibition relative to DMSO control, using Prism software, from GraphPad. Predicted IC_50_ values of compounds were then confirmed by testing again using the same assay described above. The sample size consisted of four independent trials carried out on four different days (to account for possible variation in the parasite population).

#### *In vitro* susceptibility assay against the human trichomonad *T. vaginalis* strain G3

*T. vaginalis* G3 trophozoites (from Patricia Johnson, University of California at Los Angeles, CA, USA) were maintained in TYM media (pH 6.2) at 37°C for 24 h and every 24 h, 1000 μL of cells were passed into 10 ml of TYM media to maintain the culture. To perform the initial susceptibility screens on metronidazole-sensitive *T. vaginalis* G3, compounds were dissolved in DMSO to obtain concentrations of 100 mM; 5 μL aliquots of these solutions were diluted in 5 mL of TYM medium to obtain a final concentration of 100 μM. After 24 h, cells were counted using a hemacytometer. The assays were performed in 15 mL culture tubes with *T. vaginalis* G3 strain. 0.1% DMSO-only treated parasites served as control to normalize the effects of the solvent and *in vitro* conditions. TYM media only control was also included in the assays. After 24 h, cells were counted using a hemacytometer. The IC_50_ values were determined by inoculating a constant number of parasite cells in TYM medium and running assays of increasing compound concentrations, 0.02–100 μM, and performing a regression analysis on percentage growth inhibition relative to DMSO control, using Prism software from GraphPad. Predicted IC_50_ values of compounds were then confirmed by testing again using the same assay described above. The sample size consisted of four independent trials carried out on four different days (to account for possible variation in the parasite population).

#### *G. lamblia* and *E. histolytica* IC_50_ assays

Axenic trophozoites of metronidazole-sensitive *G. lamblia* WB and *E. histolytica* HM1:IMSS were grown in TYI-S-33 medium supplemented with penicillin (100 U/mL) and streptomycin (100 μg/mL). (Diamod et al., 1978; Keister, 1983) For anti-*E. histolytica* and anti-*G. lamblia* IC_50_assays, 10 mM stocks of the test compounds were serially diluted in DMSO to achieve a concentration range of 10 mM−78 μM. 0.5 microliter of compound from this concentration range was transferred in triplicate to each well of 96-well plates and 5,000 trophozoites/well were added in a final volume of 100 μL/well. Cultures were grown for 2 days at 37°C under anaerobic conditions (GasPak EZ Anaerobe Gas Generating Pouch System (VWR). Cell growth and viability were determined by adding Cell Titer-Glo cell viability assay reagent (Promega) and measuring ATP-dependent luminescence in a microplate reader. Percent inhibition relative to maximum and minimum reference signal controls was calculated using the formula:

% Inhibition = [(mean of Maximum Signal Reference Control—Experimental Value)/(mean of Maximum Signal Reference Control—mean of Minimum Signal Reference Control)] × 100.

The 50% inhibitory concentration (IC_50_) and standard error (SE) was derived from the concentration-response curves using Prism software (GraphPad).

#### Anti-bacterial and anti-fungal susceptibility analyses

To determine if the compound library had other antimicrobial activities, several fungal and bacterial species were analyzed for susceptibility to these compounds. Antifungal activity of compounds was tested in the filamentous fungal pathogen *Aspergillus parasiticus* 5862 (National Center for Agricultural Utilization and Research, USDA-ARS, Peoria, IL, USA) and the model yeast *Saccharomyces cerevisiae* BY4741 wild type (*Mat* a *his3*Δ*1 leu2*Δ*0met15*Δ*0ura3*Δ*0*) (Open Biosystems, Huntsville, AL, USA). *A. parasiticus* was cultured at 35°C on potato dextrose agar (PDA), and *S. cerevisiae* was grown on Synthetic Glucose (SG; Yeast nitrogen base without amino acids 0.67%, glucose 2% with appropriate supplements: uracil 0.02 mg/mL, amino acids 0.03 mg/mL) or Yeast Peptone Dextrose (YPD; Bacto yeast extract 1%, Bacto peptone 2%, glucose 2%) medium at 30°C. All chemicals for culturing fungi were procured from Sigma Co. (St. Louis, MO, USA).

To evaluate antifungal activity of compounds in *A. parasiticus*, bioassays were performed in microtiter plates (triplicate wells) (3 × 10^4^ to 5 × 10^4^ CFU/mL) with RPMI 1640 medium (Sigma Co., St. Louis, MO, USA). Compounds were tested at 500 μM, where fungal growth was monitored at 24–48 h after inoculation. A numerical score from 0 to 4 was provided to each well according to the protocol outlined by the Clinical and Laboratory Standards Institute (CLSI) M38-A2 (CLSI, 2008) as follows: 0 = optically clear/no visible growth, 1 = slight growth (25% of no treatment control), 2 = prominent growth reduction (50% of no treatment control), 3 = slight growth reduction (75% of no treatment control), 4 = no growth reduction. To test antifungal activity of compounds in *S. cerevisiae*, bioassays were performed in SG liquid medium (triplicate wells in microtiter plates). Compounds were examined at 500 μM, where antifungal activity was assessed 24–48 h after inoculation. A numerical score from 0 to 4 (See above) was provided to each well by the modified protocols outlined by European Committee on Antimicrobial Susceptibility Testing (EUCAST) (Keister, 1983).

For anti-bacterial susceptibility testing, disc diffusion methods were used. Vehicle control (DMSO) and 100 mM stock compounds were diluted to 100 μM in media and incubated with empty BDL-sensi-discs (6 mm) for 20 min at room temperature. These discs were placed upon plates streaked either with *Lactobacillus reuteri* (ATCC 23272), *Lactobacillus acidophilus* (ATCC 43560), *Lactobacillus rhamnosus* (ATCC 53103), *Listeria monocytogenes* 10403 (RM2194), *Salmonella enterica* pGFP, or *Escherichia coli* K-12 MG 1655. Additionally, various antibiotic discs [levofloxacin (5 μg), gentamicin (10 μg), and gentamicin (120 μg)] were placed upon these plates as controls for sensitivity. Plates were streaked for growth of *Lactobacilli*, grown in Lactobacilli MRS at 37°C under anaerobic conditions whereas the rest of the strains was grown at 37°C aerobically in Luria Broth or Brain Heart Infusion Broth. Zones of inhibition measured in millimeters were measured for each disc.

## Results and discussion

### Synthetic chemistry

**Scheme 1:**
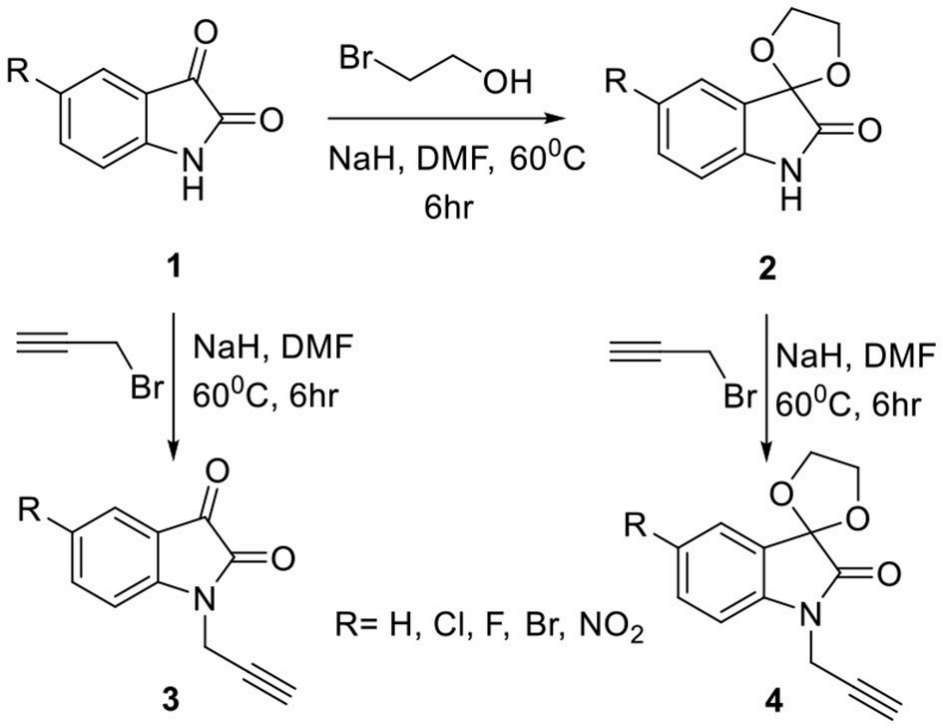
Synthesis of *N*-propargylated C-5 substituted isatin 3 and spiroketal *N*-propargylated C-5 substituted isatin 4.

**Scheme 2:**
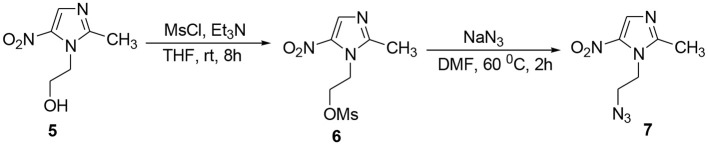
Synthesis of *N*-alkylazido-metronidazole 7.

**Scheme 3:**
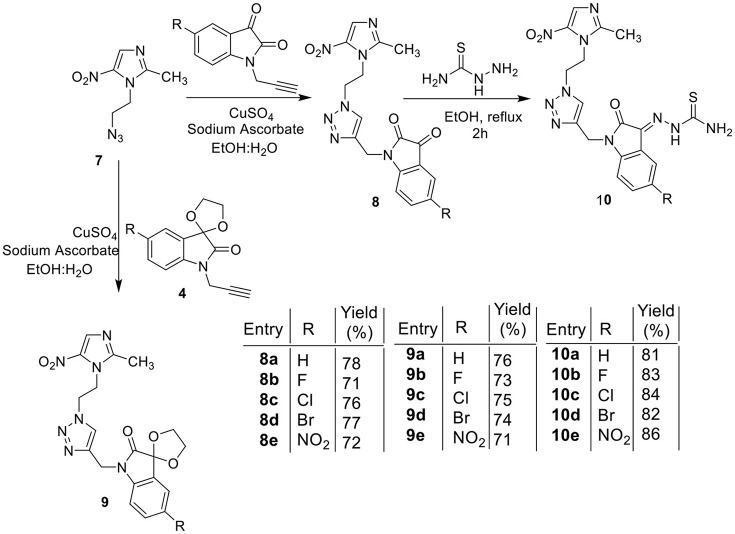
Synthesis of isatin-metronidazole conjugates 8, 9, and 10.

The methodology for the synthesis of isatin-spiroketal **2** involved sodium hydride promoted reaction of C-5 substituted isatin **1** with 1-bromoethanol (Sigma-Aldrich, Cat No. 48874, CAS No. 540-51-2) in dry DMF. *N*-propargylated C-5 substituted isatin and isatin-spiroketal were obtained via base mediated reaction of isatin/spiroisatin with propargyl bromide (Sigma-Aldrich, 80% weight in toluene, Cat No. P51001, CAS No. 106-96-7) in dry DMF at 60°C (Scheme 1). The precursor viz. 1-(2-azidoethyl)-2-methyl-5-nitro-1-*H*-imidazole **7**, was synthesized via initial mesylation of metronidazole 5 (Sigma, St. Louis, MO, USA, Cat. No. M3761) in dry THF at 0°C to form the corresponding methane-sulfonic acid 2-(2-methyl-5-nitro-imidazol-1-yl)-ethyl ester 6 followed by its nucleophilic substitution reaction with sodium azide in dry DMF at 60°C to afford the corresponding precursor 1-(2-Azido-ethyl)-2-methyl-5-nitro-1*H*-imidazole **7** in good yields (Scheme 2).Cu promoted azide-alkyne cycloaddition reaction of **7** with *N*-propargylated-isatin **3** and *N*-propargylatedspiro-isatin 4 led to the isolation of desired conjugates **8** and **9** (Scheme 3). Conjugate **8** was further treated with thiosemicarbazide to obtain metronidazole-isatin-thiosemicarbazone conjugates 10. On the bases of spectral data and analytical evidence, structures were assigned to the synthesized 1*H*-1,2,3-triazole-tethered isatin-metronidazole conjugates (see Supplementary Material). The compound 8c, for example was characterized as 5-chloro-1-{1-[2-(2-methyl-5-nitro-imidazol-1-yl)-ethyl]-1*H*-[1,2,3]triazol-4-ylmethyl}-1H-indole-2,3-dione analyzed for C_17_H_14_ClN_7_O_4_ and showed molecular ion peak at m/z 416.4302 ([M+H]^+^) and 417.4318 ([M+2]^+^) in its mass spectrum. The salient feature of its (Ravaee et al., 2015) H NMR spectra include charterstick peak at δ 4.60 (t, *J* = 4.8 Hz, 2H); δ 4.75 (t, *J* = 5.8 Hz, 2H) and one singlet at 4.90 (2H) ppm corresponding to methylene group, respectively. Two singlets also appeared at δ 7.93 and 7.97 which correspond to triazole and imidazole proton (Navaneethan and Giannella, 2008). C NMR spectrum of compound **8c** exhibited the appearance of characteristic peaks at δ 182.40 and 157.90 which correspond to isatin carbonyl (C = O) and amidic carbonyl of isatin (= N-C = O). Three methylenic carbons show characteristic peaks at δ 35.3, 46.5 and 49.3 along with one single peak at 13.3 corresponding to—CH_3_ group of imidazole ring.

### *In vitro* evaluation against *T. vaginalis, T. foetus, T. foetus*-like, *G. lamblia, E. histolytica*, fungal, and bacterial species

The chemical library of metronidazole-isatin conjugates was evaluated in a general inhibitory screen against protozoal pathogen *T. vaginalis* and the percentage inhibition results at different concentrations are enlisted in Table 1. As evident most of the conjugates exhibited 100% growth inhibition at 100 μM except **8a, 8d**, and **10e** (Table 1). The potent conjugates were further evaluated for their IC_50_ against different strains of *T. vaginalis* and *T. foetus* and compared with metronidazole, (Table 1). A closer inspection of Table 1 revealed an interesting structure activity relationship with activity being dependent upon the nature of substituent at C-3 and C-5 positions of isatin ring. In case of MTZ susceptible G-3 strain, introduction of a ketal and thiosemicarbazone substituent at C-3 position improved the activity profiles as evident from conjugates 9a–e and 10a–d. Further, the presence of halogen substituent (F, Cl, Br) improved the activity profiles with the most potent conjugate **10d** (R = Br) exhibiting an IC_50_ value of 1.2 μM against G-3 strain, which is comparable to MTZ.

**Table 1:** *In vitro* activity of isatin-metronidazole conjugates against *T. vaginalis, T. foetus, T. foetus*-like pathogen, *G. lamblia*, and *E. histolytica*.

**Entry**	**% inhibition against *T. vaginalis* at 100 μM**	**IC_50_ ± S.E (μM) *T. vaginalis* and *T. foetus***			**IC_50_ ± S.E. (μM) *G. lamblia* (WB)**	**IC_50_ ± S.E. (μM) *E. histolytica* (HM1:IMSS)**
		**G3**	**C1**	**D1**	
8a	71	N.D	N.D	7.0 ± 0.1	11.1 ± 0.1	>50
8b	100	4.7 ± 0.1	2.5 ± 0.02	1.5 ± 0.1	6.7 ± 0.01	>50
8c	100	7.1 ± 0.2	7.2 ± 0.1	N.D	3 ± 0.01	15.8 ± 0.03
8d	14	N.D	N.D	12.8 ± 0.3	14.5 ± 0.05	>50
8e	100	N.D	N.D	N.D	1.6 ± 0.01	2.3 ± 0.03
9a	100	82.3 ± 0.2	4.8 ± 0.02	18.3 ± 0.3	4 ± 0.07	7.9 ± 0.02
9b	100	7.8 ± 0.04	19 ± 0.3	16.5 ± 0.2	2.1 ± 0.01	19.5 ± 0.03
9c	100	3.2 ± 0.09	1.1 ± 0.02	N.D	1.3 ± 0.01	1.6 ± 0.03
9d	100	1.4 ± 0.02	12.9 ± 0.06	18.7 ± 0.04	0.8 ± 0.01	1.5 ± 0.03
9e	100	2.4 ± 0.02	7.8 ± 0.04	0.3 ± 0.03	1.2 ± 0.01	1.2 ± 0.08
10a	100	2.6 ± 0.08	0.6 ± 0.02	0.9 ± 0.03	0.4 ± 0.01	2.5 ± 0.02
10b	100	1.4 ± 0.02	5.3 ± 0.02	4.7 ± 0.02	0.5 ± 0.05	2.3 ± 0.02
10c	100	5.2 ± 0.03	50.8 ± 0.2	11.1 ± 0.03	7.2 ± 0.07	28.7 ± 0.04
10d	100	1.2 ± 0.04	2.0 ± 0.04	44.0 ± 0.08	5.9 ± 0.02	ND
10e	41	N.D	N.D	N.D	16.2 ± 0.08	>50
MTZ			0.72		6.4	5

*N.D., not determined*.

**Figure 3:**
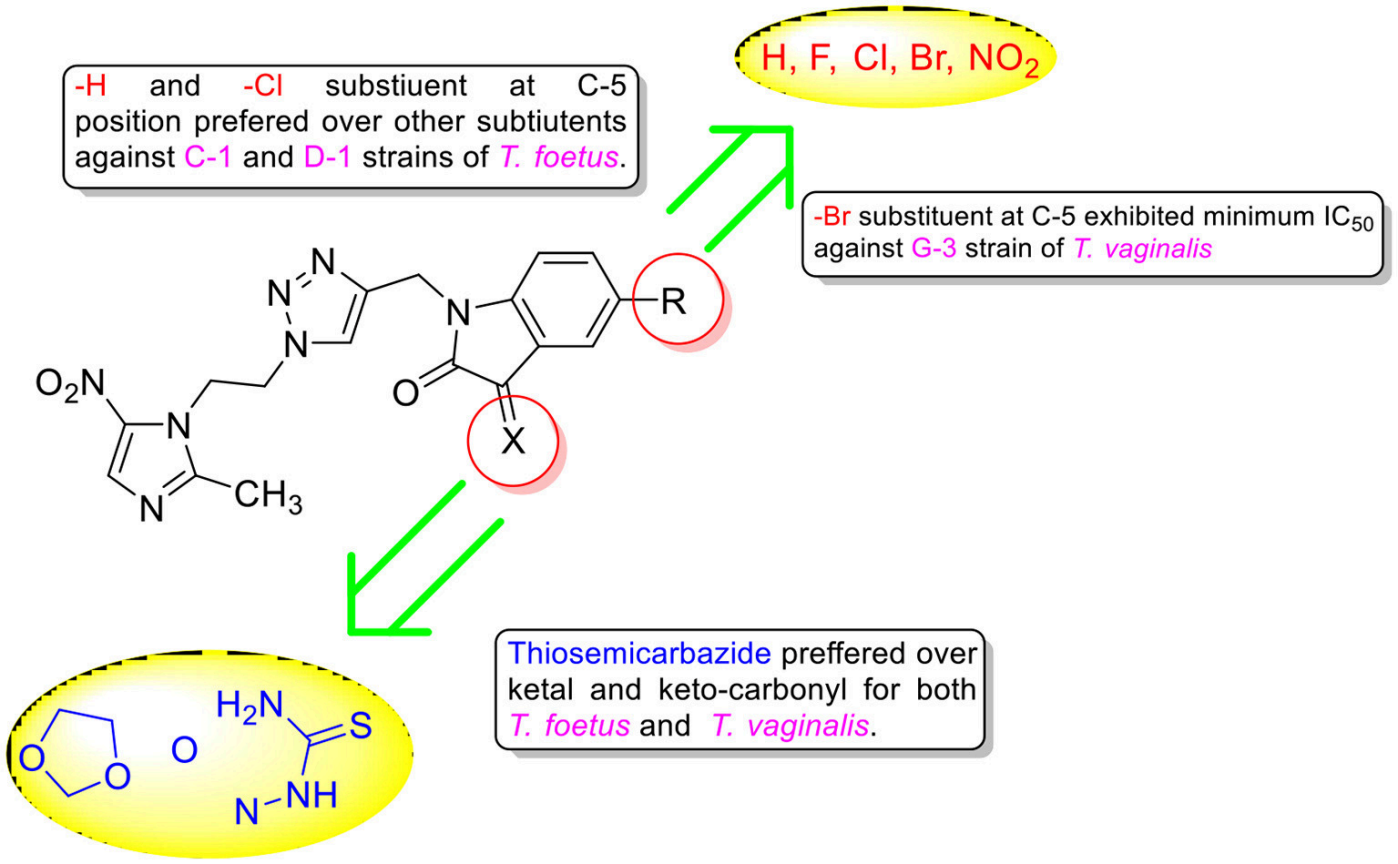
Generalized SAR of the synthesized compounds against *T. vaginalis* and *T. foetus*.

Similar SAR has been observed against C1 strain of *T. foetus*-like pathogen with activity mainly dependent upon the nature of substituent at C-3 position. The replacement of ketocarbonyl with ketal in general improved the activity profiles with conjugate **9c** (R = Cl) exhibiting an IC_50_ value of 1.1 μM. However, the introduction of thiosemicarbazide functionality substantially improved the efficacy of the conjugates with compound **10a** (R = H) displaying an IC_50_ value of 0.6 μM, better than the standard drug MTZ. The synthesized conjugates were also evaluated against D1 strain of *T. foetus*. The conjugate **10a** again proved to be the most potent among the series, exhibiting an IC_50_ value of 0.9 μM. The generalized SAR of the synthesized conjugates against *T. vaginalis* and *T. foetus* has been provided in Figure 3.

**Figure 4:**
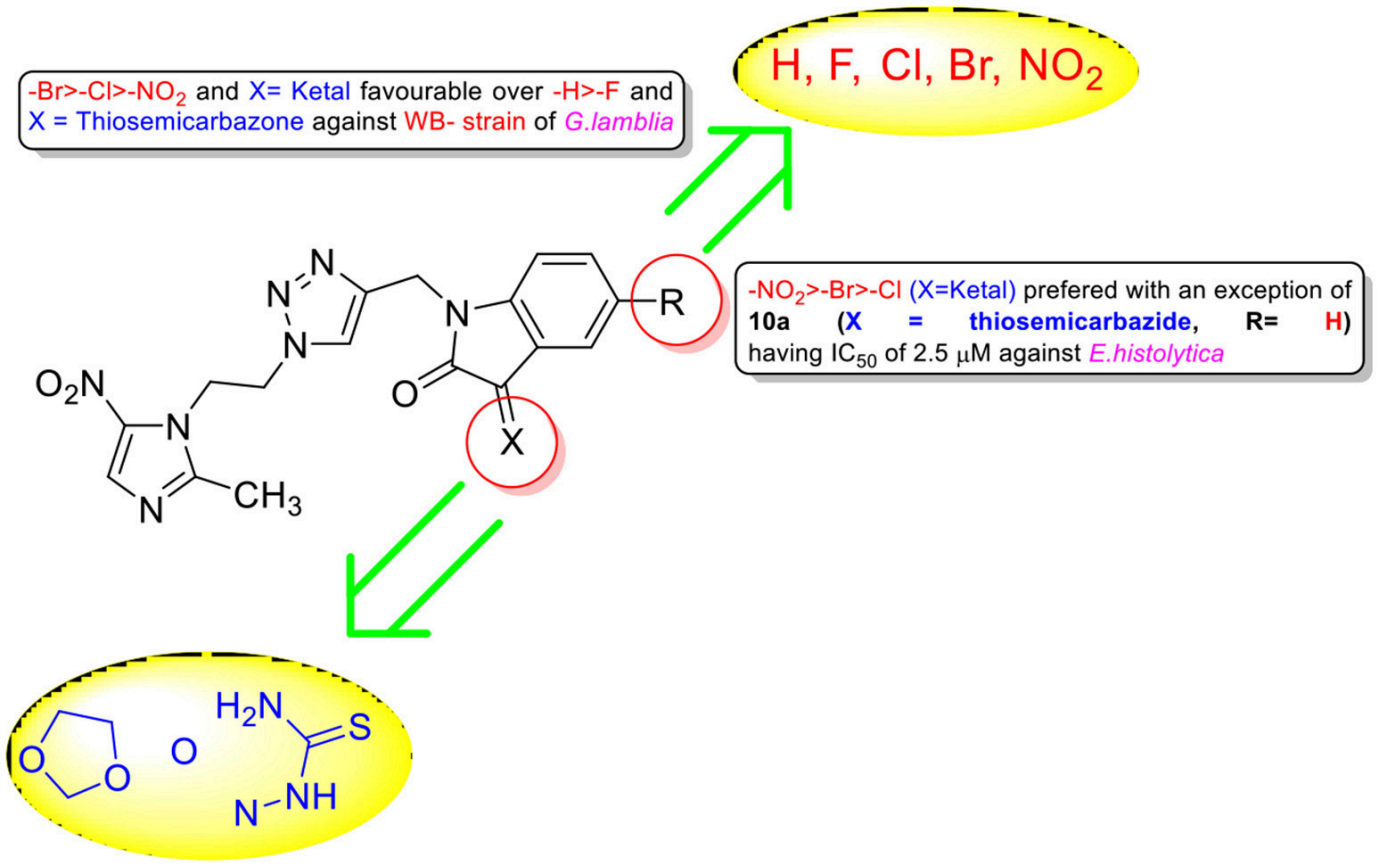
Generalized SAR of the synthesized compounds against *G. lamblia* and *E. histolytica*.

Encouraged with these biological results, it was considered worthwhile to examine the activities of synthesized conjugates against other anaerobic protozoans such as WB strain of *G. lamblia* and HM1 strain of *E. histolytica*. Since trophozoites are the relevant forms that cause trichomoniasis, amebiasis and giardiasis, we tested the activity of compounds against trophozoite forms. As evident from IC_50_ values enlisted in Table 1, all conjugates exhibited good activity profiles with IC_50_ values ranging from 0.4 to 16.2 μM against WB strain of *G. lamblia*. Activity profiles showed a preference for electron withdrawing substituent (R = F, Cl, Br, NO_2_) at C-5 position of isatin except **10a** (R = H) which exhibited an IC_50_ value of 0.4 μM. The replacement of ketocarbonyl with ketal and thiosemicarbazide improved the activities except **8e** which displayed an IC_50_ value of 1.6 μM. Conjugates **8c, 8e, 9a, 9b, 9c, 9d, 9e, 10a, 10b**, and **10d** proved to be more potent than the standard drug MTZ (IC_50_ = 6.4 μM) (Arendrup et al., 2012), with conjugates **9d, 10a, and 10b** exhibiting IC_50_ values in sub-micromolar concentration. Similarly, conjugates **8e, 9c, 9d, 9e, 10a**, and **10b** showed better potency than the current standard of care MTZ (IC_50_ = 5 μM)(Bashyal et al., 2017) against *E. histolytica* (Table 1). The generalized SAR of the synthesized conjugates against *E. histolytica* and *G. lamblia* has been provided in Figure 4.

To determine if these compounds were specific for protozoal parasites, we also tested a number of different fungal and bacterial species. In all assays, the activities of these compounds were specific for protozoa and exerted no detectable antifungal and antibacterial properties.

In conclusion, the present study undertook the synthesis of 1*H*-1,2,3-triazole-tethered metronidazole-isatin conjugates and evaluated their activity against multiple protozoan parasites, namely *T. vaginalis, T. foetus, T. foetus*-like pathogen, *G. lamblia* and *E. histolytica*. SAR studies revealed the dependence of activity profiles on the nature of substituent at C-3 and C-5 positions of isatin ring with most of the synthesized conjugates exhibiting comparable efficacies to that of MTZ against different trichomonal strains while better inhibitory activities were observed against *G. lamblia*. Interestingly, introduction of thiosemicarbazide into the basic core of the synthesized conjugates improved the activities with most potent conjugate **10a** being ~16 folds more active than MTZ against *G. lamblia*, ~2 folds more active than MTZ against *E. histolytica* and equipotent to MTZ against *T. vaginalis* and *T. foetus*. Further work on improving the activities of most promising conjugates of the synthesized library, is presently underway.

## Author contributions

SK synthesized and characterized metronidazole-isatin, metronidazole-spiroisatin, and metronidazole-isatin-thiosemicarbazones. TB performed *E. histolytica* and *G. lamblia* experiments. AD conceptualized the *E. histolytica* and *G. lamblia* studies, analyzed the data, reviewed, and edited the manuscript. KL conceptualized the *T. vaginalis* and *T. foetus*, and *T. foetus*-like pathogen studies, analyzed the data, reviewed, and edited the manuscript. AW performed *T. vaginalis, T. foetus*, and *T. foetus*-like pathogen experiments. LC conceptualized the anti-fungal and anti-bacterial studies, analyzed the data, reviewed, and edited the manuscript. JK and CT performed the anti-fungal and anti-bacterial experiments, respectively. VK designed and characterized the described conjugates, reviewed, and edited the manuscript.

### Conflict of interest statement

The authors declare that the research was conducted in the absence of any commercial or financial relationships that could be construed as a potential conflict of interest.

## Supplementary material

Supplementary Data Sheet 1 ^1^H, ^13^C NMR data of all the synthesized compounds along with scanned ^1^H, ^13^C and ^13^C (DEPT) NMR of 8a, 8b, 8c, 9c, 10a, 10b.
