# Supplementary material for: Highly Potent 1H-1,2,3-Triazole-Tethered Isatin-Metronidazole Conjugates Against Anaerobic Foodborne, Waterborne, and Sexually-Transmitted Protozoal Parasites
Source: Front Cell Infect Microbiol. 2018 Oct 30;8:380. doi: 10.3389/fcimb.2018.00380 (PMC6218680; doi:10.3389/fcimb.2018.00380)

Highly potent 1*H*-1,2,3-triazole-tethered isatin-metronidazole conjugates against anaerobic foodborne, waterborne, and sexually-transmitted protozoal parasites

Sumit Kumar^1^, Trpta Bains^2^, Ashley Sae Won Kim,^4^ Christina Tam^3^, Jong Kim^3^,

Luisa W. Cheng^3^, Kirkwood M. Land^4^, Anjan Debnath^2*^, Vipan Kumar^1*^

^1^Department of Chemistry, Guru Nanak Dev University, Amritsar.143005, India.

^2^Center for Discovery and Innovation in Parasitic Diseases, Skaggs School of Pharmacy and Pharmaceutical Sciences, University of California San Diego, La Jolla, United States.

^3^Foodborne Toxin Detection and Prevention Research Unit, Agricultural Research Service, United States Department of Agriculture, Albany CA.

^4^Department of Biological Sciences, University of the Pacific, 3601 Pacific Avenue, Stockton, CA 95211.

**1-((1-(2-(2-methyl-5nitro-1H-imidazol-1-yl)ethyl)-1H-1,2,3-triazol-4-yl)methyl)indoline-2,3-dione (8a)**: Yield 78 %, orange solid, mp 166-167 ^0^C; ^1^HNMR (500 MHz, DMSO-*d*_6_) δ 1.73 (s, 3H, -CH_3_); 4.64 (t, *J*=4.4 Hz, 2H, -CH_2_), 4.78 (t, *J*=5.4 Hz, 2H, -CH_2_), 4.93 (s, 2H, -CH_2_), 7.11-7.13 (m, 2H, Ar-H), 7.48-7.53 (m, 2H, Ar-H), 7.95 (s, 1H, triazole-H), 7.99 (s, 1H, imidazole-H). ^13^C NMR (100 MHz, DMSO-d_6_) δ 13.2, 35.2, 46.5, 49.2, 111.9, 113.0, 118.9, 124.4, 124.6, 125.0, 133.5, 138.7, 142.1, 146.6, 151.6, 158.1, 182.9; HRMS calcd for C_17_H_15_N_7_O_4_ [M+H]^+^ 382.1219 found 382.1208.

**5-fluoro-1-((1-(2-(2-methyl-5nitro-1H-imidazol-1-yl)ethyl)-1H-1,2,3-triazol-4-yl)methyl)indoline-2,3-dione (8b)**:

Yield 71 %, red solid, mp 155-156 ^0^C; ^1^HNMR (500 MHz, DMSO-*d*_6_) δ 1.75 (s, 3H, -CH_3_); 4.65 (t, *J*=4.8 Hz, 2H, -CH_2_), 4.78 (t, *J*=5.6 Hz, 2H, -CH_2_), 4.93 (s, 2H, -CH_2_ ), 7.13(d, *J*=8.5 Hz, 1H, Ar-H), 7.63-7.70 (m, 2H, Ar-H), 7.95 (s, 1H, triazole-H), 7.99 (s, 1H, imidazole-H). ^13^C NMR (100 MHz, DMSO-d_6_) δ 13.2, 35.3, 46.4, 49.2, 113.3, 119.3, 124.4, 125.0, 128.1, 133.5, 137.5, 138.7, 142.0, 148.9, 151.6, 157.9, 182.4; HRMS calcd for C_17_H_14_FN_7_O_4_ [M+H]^+^ 400.1125 found 400. 1134;

**5-chloro-1-((1-(2-(2-methyl-5nitro-1H-imidazol-1-yl)ethyl)-1H-1,2,3-triazol-4-yl)methyl)indoline-2,3-dione (8c)**:

Yield 76 %, orange solid, mp 169-170 ^0^C; ^1^HNMR (400 MHz, DMSO-*d*_6_) δ 1.69 (s, 3H, -CH_3_); 4.60 (t, *J*=4.8 Hz, 2H, -CH_2_); 4.75 (t, *J*=5.8 Hz, 2H, -CH_2_); 4.90 (s, 2H, -CH_2_); 7.10 (d, *J*=8.4 Hz, 1H, Ar-H); 7.60-7.67 (m, 2H, Ar-H); 7.93 (s, 1H, triazole-H); 7.97 (s, 1H, imidazole-H) ^13^C NMR (100 MHz, DMSO-d_6_) δ 13.3, 35.3, 46.5, 49.3, 113.3, 119.4, 124.5, 125.1, 128.1, 133.6, 137.5, 138.8, 142.1, 148.9, 151.6, 157.9, 182.4; HRMS calcd for C_17_H_14_ClN_7_O_4_ [M+H]^+^ and [M+2]^+^ 416.0876 and 417.0766 found 416.4302 and 417.4318.

**5-bromo-1-((1-(2-(2-methyl-5nitro-1H-imidazol-1-yl)ethyl)-1H-1,2,3-triazol-4-yl)methyl)indoline-2,3-dione (8d)**:

Yield 77%, buff red solid, mp 162-163 ^0^C; ^1^HNMR (400 MHz, DMSO-*d*_6_) δ 1.67 (s, 3H, -CH_3_), 4.63 (t, *J*=4.6 Hz, 2H, -CH_2_), 4.73 (t, *J*=5.6 Hz, 2H, -CH_2_), 4.91 (s, 2H, -CH_2_), 7.13 (d, *J*=8.4 Hz, 1H, Ar-H), 7.62-7.68 (m, 2H, Ar-H), 7.94 (s, 1H, triazole-H), 7.96 (s, 1H, imidazole-H) ^13^C NMR (100 MHz, DMSO-d_6_) δ 13.1, 35.4, 46.6, 49.4, 112.4, 119.2, 124.7, 125.2, 128.5, 133.2, 137.9, 138.7, 142.2, 148.4, 151.7, 157.8, 182.3; HRMS calcd for C_17_H_14_BrN_7_O_4_ [M+H]^+^ 461.0270 found 461.0282.

**5-nitro-1-((1-(2-(2-methyl-5nitro-1H-imidazol-1-yl)ethyl)-1H-1,2,3-triazol-4-yl)methyl)indoline-2,3-dione (8e):**

Yield 72 %, yellow solid, 170-171 ^0^C; ^1^HNMR (400 MHz, DMSO-*d*_6_) δ 1.63 (s, 3H, -CH_3_), 4.61 (t, *J*=4.8 Hz, 2H, -CH_2_), 4.74 (t, *J*=5.8 Hz, 2H, -CH_2_), 4.91 (s, 2H, -CH_2_), 7.09 (d, *J*=8.4 Hz, 1H, Ar-H), 7.62-7.69 (m, 2H, Ar-H), 7.95 (s, 1H, triazole-H), 7.97 (s, 1H, imidazole-H) ^13^C NMR (100 MHz, DMSO-d_6_) δ 13.3, 35.7, 46.2, 49.8, 113.4, 119.3, 124.9, 125.4, 128.2, 133.7, 137.4, 138.4, 142.3, 148.7, 151.4, 157.8, 182.1; HRMS calcd for C_17_H_14_N_8_O_6_ [M+H]^+^ 427.1070 found 427.1062.

**1-((1-(2-(2-methyl-5-nitro-1H-imidazol-1-yl)ethyl)-1*H*-1,2,3-triazol-4-yl)methyl)spiro[indoline-3,2’-[1,3]dioxolqn]-2-one (9a):**

Yield 76%, light brown solid, 158-159 ^0^C; ^1^HNMR (500 MHz, DMSO-*d*_6_) δ 1.72, (s, 3H, -CH_3_), 4.29-4.37 (m, 4H, 2x-CH_2_), 4.66 (t, *J*=4.6 Hz, 2H, -CH_2_) 4.77 (t, *J*=5.4 Hz, 2H, -CH_2_); 4.94 (s, 2H, -CH_2_); 7.12-7.14 (m, 2H, Ar-H), 7.49-7.57 (m, 2H, Ar-H),); 7.91 (s, 1H, triazole-H); 7.93 (s, 1H, imidazole-H) ^13^C NMR (100 MHz, DMSO-d_6_) 13.1, 34.1, 46.2, 49.2, 66.6, 101.2, 112.4, 124.3, 125.9, 126.6, 127.2, 131.8, 134.1, 137.5, 142.2, 142.4, 151.1, 172.8; HRMS calcd for C_19_H_19_N_7_O_5_ [M+H]^+^ 426.1481 found 426.4625.

**5-fluoro-1-((1-(2-(2-methyl-5-nitro-1H-imidazol-1-yl)ethyl)-1*H*-1,2,3-triazol-4-yl)methyl)spiro[indoline-3,2’-[1,3]dioxolqn]-2-one(9b):**

Yield 73% red solid 163-164 ^0^C; ^1^HNMR (500 MHz, DMSO-*d*_6_) δ 1.71 (s, 3H, -CH_3_); 4.29-4.36 (m, 4H, 2x-CH_2_); 4.68 (t, *J*=4.8 Hz,2H, -CH_2_); 4.79 (t, *J*=5.4 Hz, 2H, -CH_2_); 4.86 (s, 2H, -CH_2_); 7.04 (d, *J*=8.2 Hz, 1H, Ar-H); 7.36-7.43 (m, 2H, Ar-H); 7.97 (s, 1H, triazole-H); 7.99 (s, 1H, imidazole-H) ^13^C NMR (100 MHz, DMSO-d_6_) 13.3, 34.7, 46.5, 49.1, 66.3, 101.5, 112.7, 124.3, 125.3, 126.2, 127.4, 131.2, 133.4, 138.5, 142.1, 142.3, 151.7, 172.4; HRMS calcd for C_19_H_18_FN_7_O_5_ [M+H]^+^ 444.1387 found 444. 1393.

**5-chloro-1-((1-(2-(2-methyl-5-nitro-1H-imidazol-1-yl)ethyl)-1*H*-1,2,3-triazol-4-yl)methyl)spiro[indoline-3,2’-[1,3]dioxolqn]-2-one (9c):**

Yield 75%, light red solid, 171-172 ^0^C; ^1^HNMR (400 MHz, DMSO-*d*_6_) δ 1.66 (s, 3H, -CH_3_); 4.24-4.33 (m, 4H, 2x-CH_2_), 4.62 (t, *J*=4.6 Hz,2H, -CH_2_), 4.75 (t, *J*=5.4 Hz, 2H, -CH_2_), 4.82 (s, 2H, -CH_2_), 7.01 (d, *J*=8.3 Hz, 1H, Ar-H), 7.42-7.45 (m, 2H, Ar-H), 7.96 (s, 1H, triazole-H), 7.98 (s, 1H, imidazole-H) ^13^C NMR (100 MHz, DMSO-d_6_) 13.2, 34.9, 46.6, 49.3, 66.4, 101.5, 112.2, 124.9, 125.3, 126.6, 127.8, 131.8, 133.7, 138.8, 142.1, 142.2, 151.4, 172.2; HRMS calcd for C_19_H_18_ClN_7_O_5_ [M+H]^+^ and [M+2]^+^ 460.1138 and 461.1028 found 460.1158 and 461.1043.

**5-bromo-1-((1-(2-(2-methyl-5-nitro-1H-imidazol-1-yl)ethyl)-1*H*-1,2,3-triazol-4-yl)methyl)spiro[indoline-3,2’-[1,3]dioxolqn]-2-one (9d):**

Yield 74 %, light cream solid, 166-167 ^0^C; ^1^H NMR (500 MHz, DMSO-*d*_6_) δ 1.68 (s, 3H, -CH_3_), 4.26-4.35 (m, 4H, 2x-CH_2_), 4.68 (t, *J*=4.4 Hz, 2H, -CH_2_), 4.81 (t, *J*=5.4 Hz, 2H, -CH_2_), 4.99 (s, 2H, -CH_2_), 7.12 (d, *J*=8.2 Hz, 1H, Ar-H), 7.59-7.67 (m, 2H, Ar-H), 7.95 (s, 1H, triazole-H), 7.97 (s, 1H, imidazole-H) ^13^C NMR (500 MHz, DMSO-*d*_6_) δ 13.1, 34.6, 46.2, 49.8, 66.8, 101.3, 112.8, 124.4, 125.8, 126.4, 127.6, 131.3, 133.6, 138.7, 142.1, 142.3, 151.7, 172.3; HRMS calcd for C_19_H_18_BrN_7_O_5_ [M+H]^+^ 505.0532 found 505.0526.

**5-nitro-1-((1-(2-(2-methyl-5-nitro-1H-imidazol-1-yl)ethyl)-1*H*-1,2,3-triazol-4-yl)methyl)spiro[indoline-3,2’-[1,3]dioxolqn]-2-one (9e):**

Yield 71%, light yellow solid, 171-172 ^0^C; ^1^HNMR (500 MHz, DMSO-*d*_6_) δ 1.64 (s, 3H, -CH_3_), 4.26-4.33 (m, 4H, 2x-CH_2_), 4.66 (t, *J*=4.4 Hz,2H, -CH_2_), 4.79 (t, *J*=5.6 Hz, 2H, -CH_2_), 4.91 (s, 2H, -CH_2_), 7.09 (d, *J*=8.2 Hz, 1H, Ar-H), 7.51-7.59 (m, 2H, Ar-H), 7.94 (s, 1H, triazole-H), 7.96 (s, 1H, imidazole-H) ^13^C NMR (100 MHz, DMSO-d_6_) 13.4, 34.1, 46.8, 49.2, 66.4, 101.9, 112.4, 124.1, 125.2, 126.7, 127.1, 131.4, 133.2, 138.3, 142.1, 142.3, 151.6, 171.9; HRMS calcd for C_19_H_18_N_8_O_7_ [M+H]^+^ 471.1332 found 471.1341.

**(*E*)-2-((1-(2-(2-methyl-5-nitro-1*H*-imidazol-1-yl)ethyl)-1*H*-1,2,3-triazol-4-yl)methyl)-2-oxoindolin-3-ylidene)hydrazine-1-carbothioamide (10a):**Yeild 81 %, light yellow solid, 178-179 ^0^C; ^1^HNMR (300 MHz, DMSO-*d*_6_) δ 1.60 (s, 3H, -CH_3_), 4.59 (t, *J*= 4.3 Hz, 2H, -CH_2_), 4.74 (t, *J*= 5.4 Hz, 2H, -CH_2_), 4.95 (s, 2H, -CH_2_), 7.09-7.23 (m, 2H, Ar-H), 7.52 (dd, *J_1,3_* = 7.8, *J*_1,2_ = 2.3 Hz, 2H, Ar-H), 7.93 (s, 1H, triazole-H), 7.94 (s, 1H, imidazole-H), 8.80 (s, 1H, -NH-, exchangeable with D_2_O), 9.16 (s, 1H, -NH-, exchangeable with D_2_O), 12.21 (s, 1H, -NH-, exchangeable with D_2_O) ^13^C NMR (100 MHz, DMSO-*d*_6_) δ 13.0, 34.7, 46.6, 49.3, 110.6, 119.7, 121.3, 123.5, 125.1, 131.3, 131.5, 133.4, 138.6, 142.3, 142.5, 151.5, 160.0, 179.2; HRMS calcd for C_18_H_18_N_10_O_3_S [M+Na]^+^ 477.1318 found 477.1357.

**(*E*)-2-(5-fluoro-1-((1-(2-(2-methyl-5-nitro-1*H*-imidazol-1-yl)ethyl)-1*H*-1,2,3-triazol-4-yl)methyl)-2-oxoindolin-3-ylidene)hydrazine-1-carbothioamide (10b):**

Yield 83 %, light yellow solid, 175-176 ^0^C; ^1^HNMR (500 MHz, DMSO-*d*_6_) δ 1.67 (s, 3H, -CH_3_), 4.63 (t, *J*=4.9 Hz, 2H, -CH_2_), 4.78 (t, *J*=5.8 Hz, 2H, -CH_2_), 4.98 (s, 2H, -CH_2_), 7.10 (d, *J*=8.4 Hz, 1H, Ar-H), 7.57 (dd, *J_1,3_* = 8.4, *J_1,2_* = 2.0 Hz, 1H, Ar-H), 7.93 (d, *J*=1.9 Hz, 1H, Ar-H), 7.95 (s, 1H, triazole-H), 7.96 (s, 1H, imidazole-H), 8.88 (s, 1H, -NH-, exchangeable with D_2_O), 9.16 (s, 1H, -NH-, exchangeable with D_2_O), 12.20 (s, 1H,-NH-, exchangeable with D_2_O) ^13^C NMR (100 MHz, DMSO-*d*_6_) δ 13.1, 34.9, 46.5, 49.2, 79.5, 112.7, 115.4, 122.0, 123.7, 125.0, 129.8, 131.6, 133.5, 138.7, 141.6, 142.1, 151.5, 160.3, 179.2; HRMS calcd for C_18_H_17_FN_10_O_3_S [M+Na]^+^ 495.1190 found 495.1183.

##

**(*E*)-2-(5-chloro-1-((1-(2-(2-methyl-5-nitro-1*H*-imidazol-1-yl)ethyl)-1*H*-1,2,3-triazol-4-yl)methyl)-2-oxoindolin-3-ylidene)hydrazine-1-carbothioamide(10c):**

Yield 84%, light yellow solid, 171-172 ^0^C; ^1^HNMR (400 MHz, DMSO-*d*_6_) δ 1.61 (s, 3H, -CH_3_), 4.59 (t, *J=*4.8 Hz, 2H, -CH_2_), 4.74 (t, *J*=5.6 Hz, 2H, -CH_2_), 4.96 (s, 2H, -CH_2_), 7.11 (d, *J*=8.4 Hz, 1H, Ar-H), 7.42 (dd, *J_1,3_* = 8.4, *J_1,2_* = 2.1 Hz, 1H, Ar-H), 7.77 (d, *J*=2.0 Hz, 1H, Ar-H), 7.92 (s, 1H, triazole-H), 7.94 (s, 1H, imidazole-H), 8.85 (s, 1H, -NH-, exchangeable with D_2_O), 9.16 (s, 1H, -NH-, exchangeable with D_2_O), 12.15 (s, 1H,-NH-, exchangeable with D_2_O) ^13^C NMR (100 MHz, DMSO-*d*_6_) δ 13.2, 34.4, 46.7, 49.3, 79.7, 112.8, 114.9, 122.3, 123.2, 125.1, 128.9, 131.9, 133.4, 138.4, 141.8, 142.2, 151.6, 160.2, 179.1; HRMS calcd for C_18_H_17_ClN_10_O_3_S [M+Na]^+^  and [M+Na+2]^+^ 511.0894 and 513.0865 found 511.1002 and 513.0891.

**(*E*)-2-(5-bromo-1-((1-(2-(2-methyl-5-nitro-1*H*-imidazol-1-yl)ethyl)-1*H*-1,2,3-triazol-4-yl)methyl)-2-oxoindolin-3-ylidene)hydrazine-1-carbothioamide(10d):**

Yield 82%, light yellow solid, 177-178 ^0^C; ^1^HNMR (500 MHz, DMSO-*d*_6_) δ 1.69 (s, 3H, -CH_3_), 4.61 (t, *J*=4.6 Hz, 2H, -CH_2_), 4.79 (t, *J*=5.6 Hz, 2H, -CH_2_), 4.93 (s, 2H, -CH_2_), 7.13 (d, *J*=8.4 Hz, 1H, Ar-H), 7.58 (dd, *J_1,3_* = 8.4, *J_1,2_* = 2.0 Hz, 1H, Ar-H), 7.91 (d, *J*=2.0 Hz, 1H, Ar-H), 7.94 (s, 1H, triazole-H), 7.95 (s, 1H, imidazole-H), 8.81 (s, 1H, -NH-, exchangeable with D_2_O), 9.14 (s, 1H, -NH-, exchangeable with D_2_O), 12.18 (s, 1H,-NH-, exchangeable with D_2_O) ^13^C NMR (100 MHz, DMSO-*d*_6_) δ 13.3, 34.8, 46.9, 49.1, 79.2, 112.2, 115.4, 122.0, 123.4, 125.0, 129.7,131.7, 133.5, 138.7, 141.2, 142.1, 151.5, 160.3, 179.6; HRMS calcd for C_18_H_17_BrN_10_O_3_S [M+Na]^+^ 534.0369 found 534.0377.

**(*E*)-2-(5-nitro-1-((1-(2-(2-methyl-5-nitro-1*H*-imidazol-1-yl)ethyl)-1*H*-1,2,3-triazol-4-yl)methyl)-2-oxoindolin-3-ylidene)hydrazine-1-carbothioamide(10e):**

Yield 86%, light yellow solid, 181-182 ^0^C; ^1^HNMR (500 MHz, DMSO-*d*_6_) δ 1.69 (s, 3H, -CH_3_), 4.60 (t, *J=*4.4 Hz, 2H, -CH_2_), 4.79 (t, *J*=5.4 Hz, 2H, -CH_2_), 4.94 (s, 2H, -CH_2_), 7.09 (d, *J*=8.2 Hz, 1H, Ar-H), 7.51 (dd, *J_1,3_* = 8.2, *J_1,2_* = 2.0 Hz, 1H, Ar-H), 7.87 (d, *J*=2.0 Hz, 1H, Ar-H), 7.93 (s, 1H, triazole-H), 7.94 (s, 1H, imidazole-H), 8.86 (s, 1H, -NH-, exchangeable with D_2_O), 9.14 (s, 1H, -NH-, exchangeable with D_2_O), 12.12 (s, 1H,-NH-, exchangeable with D_2_O) ^13^C NMR (100 MHz, DMSO-*d*_6_) δ 13.1, 34.9, 46.2, 49.6, 79.6, 112.1, 114.6, 122.7, 123.6, 125.2, 128.5, 131.9, 133.6, 138.3, 141.1, 142.4, 151.4, 160.6, 179.4; HRMS calcd for C_18_H_17_N_11_O_5_S [M+Na]^+^ 522.1135 found 522.1148.

**^1^H NMR (400 MHz, DMSO-*d_6_*) of 5-chloro-1-((1-(2-(2-methyl-5-nitro-1*H*-imidazol-1-yl)ethyl)-1*H*-1,2,3-triazol-4yl)methyl)indoline-2,3-dione (8c):**


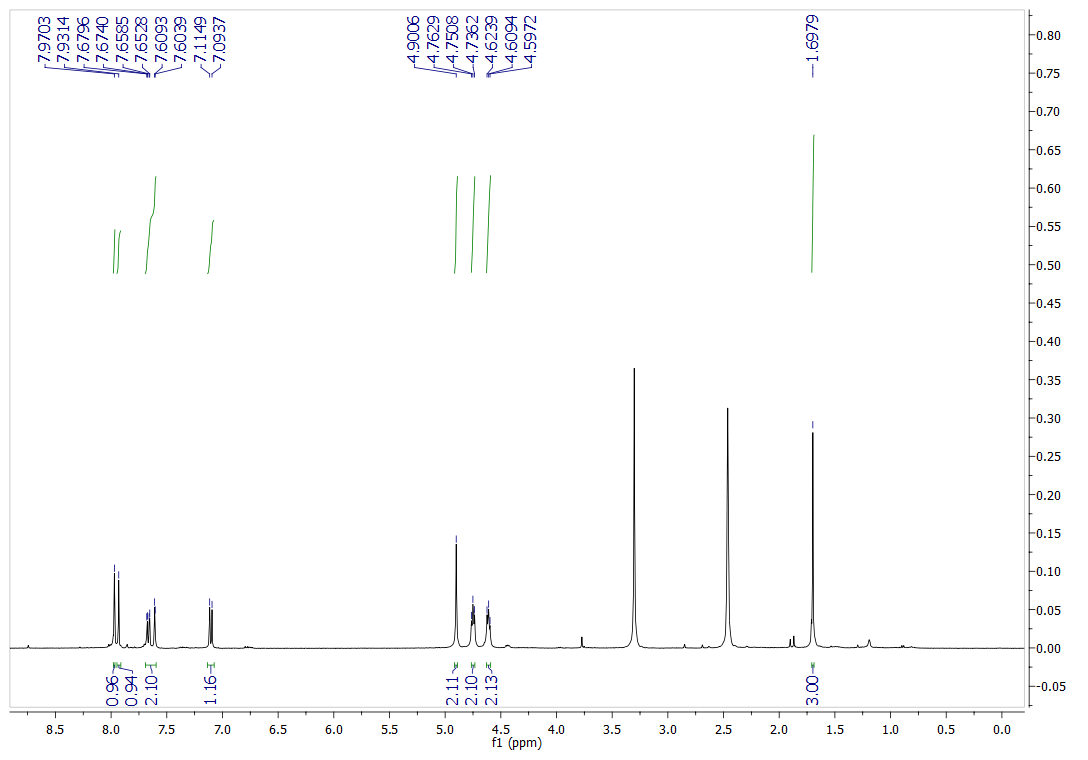


**^13^C NMR (100 MHz, DMSO-*d_6_*) of 5-chloro-1-((1-(2-(2-methyl-5-nitro-1*H*-imidazol-1-yl)ethyl)-1*H*-1,2,3-triazol-4yl)methyl)indoline-2,3-dione (8c):**


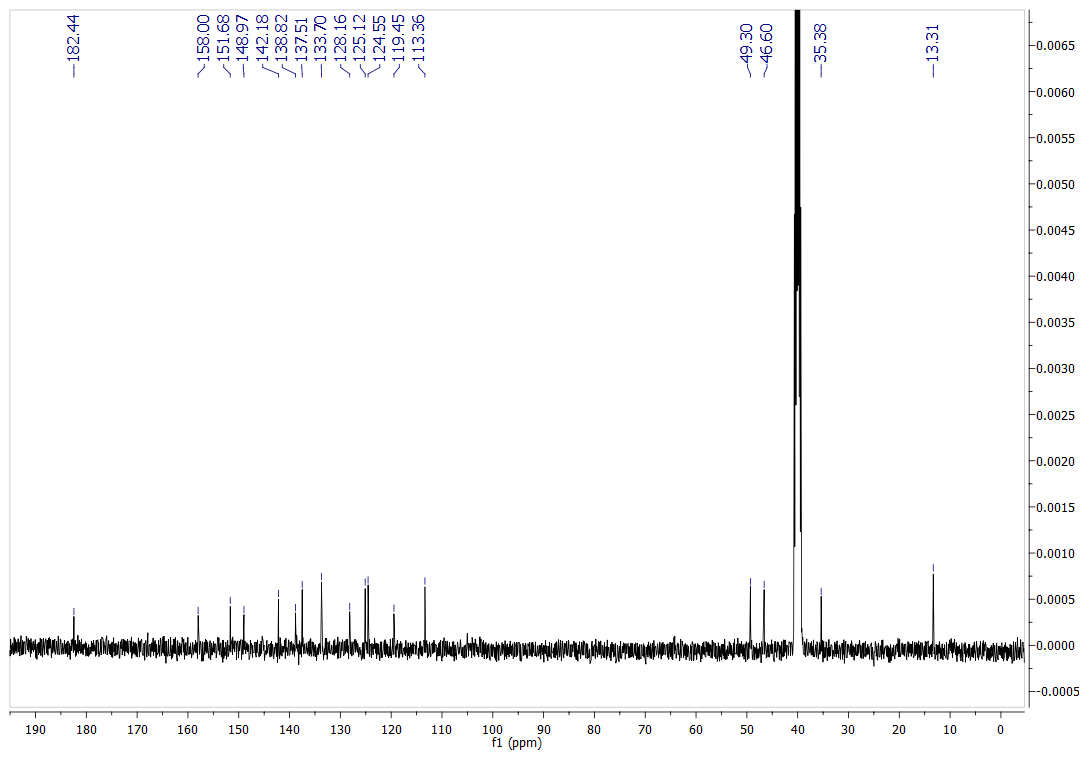


**^13^C-Dept NMR of 5-chloro-1-((1-(2-(2-methyl-5-nitro-1*H*-imidazol-1-yl)ethyl)-1*H*-1,2,3-triazol-4yl)methyl)indoline-2,3-dione (8c):**


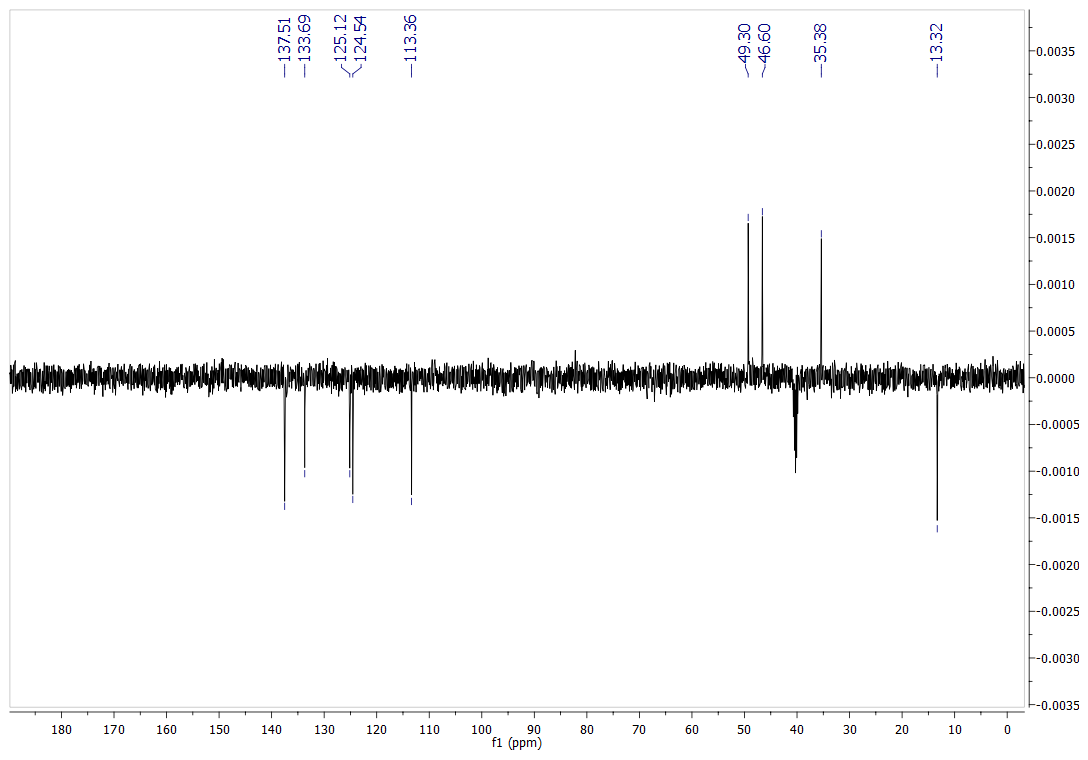


**^1^H NMR (400 MHz, DMSO-*d_6_*) of 5-chloro-1-((1-(2-(2-methyl-5-nitro-1*H*-imidazol-1-yl)ethyl)-1*H*-1,2,3-triazol-4-yl)methyl)spiro[indoline-3,2’-[1,3]dioxolan]-2-one (9c):**


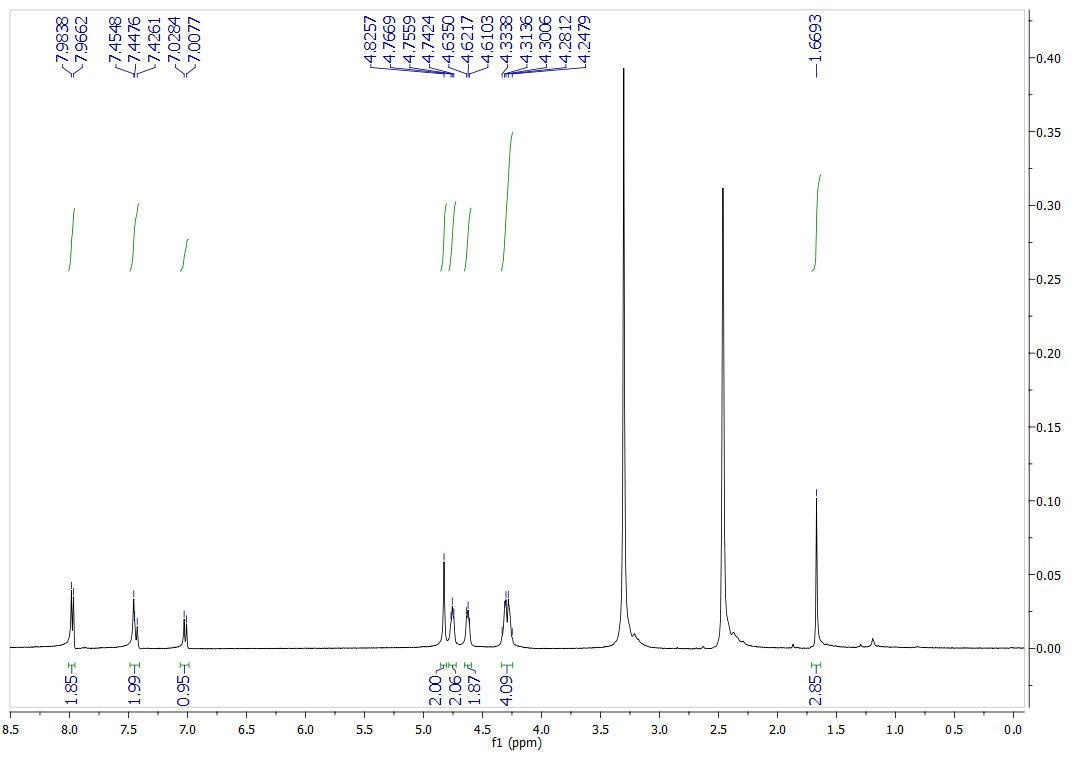


**^13^C NMR (100 MHz, DMSO-*d_6_*) of 5-chloro-1-((1-(2-(2-methyl-5-nitro-1*H*-imidazol-1-yl)ethyl)-1*H*-1,2,3-triazol-4-yl)methyl)spiro[indoline-3,2’-[1,3]dioxolan]-2-one (9c):**


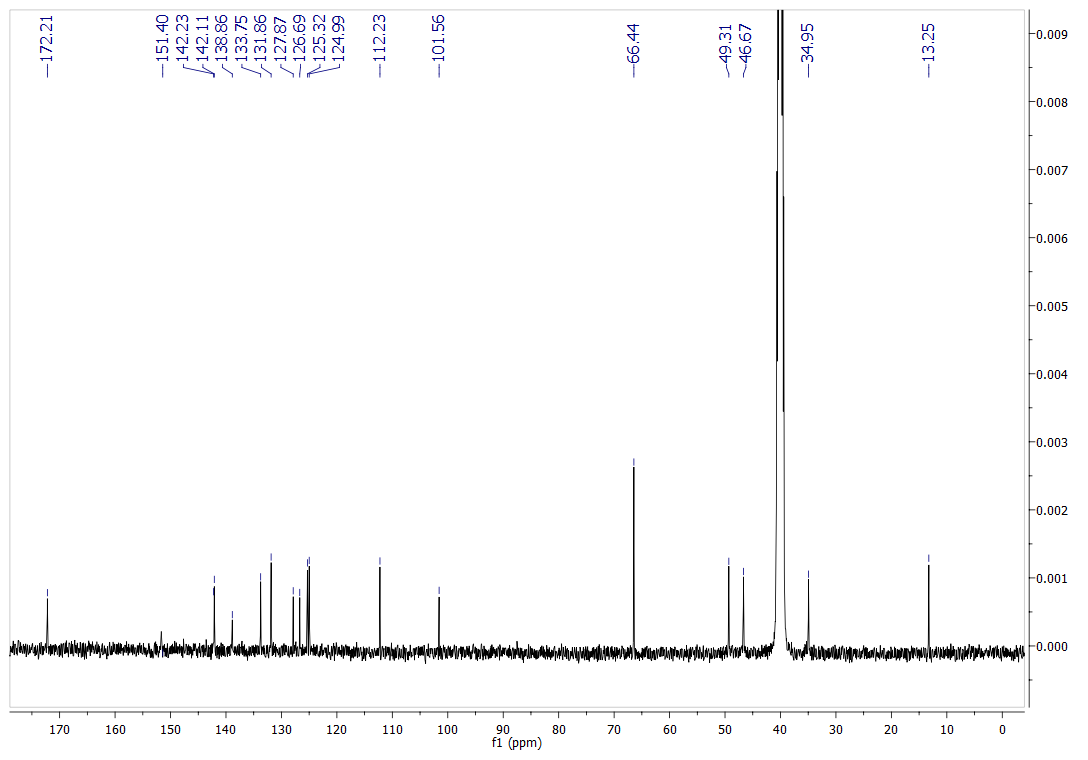


**^13^C-Dept NMR (100 MHz, DMSO-*d_6_*) of 5-chloro-1-((1-(2-(2-methyl-5-nitro-1*H*-imidazol-1-yl)ethyl)-1*H*-1,2,3-triazol-4-yl)methyl)spiro[indoline-3,2’-[1,3]dioxolan]-2-one (9c):**


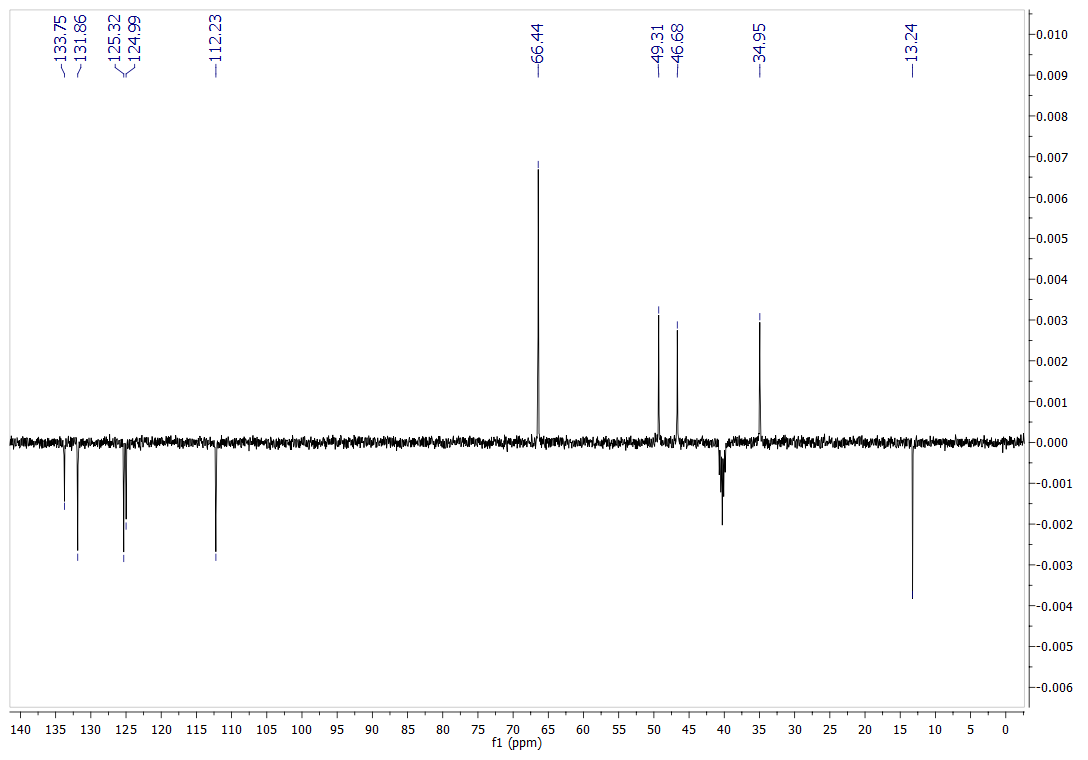


**^1^H NMR (500 MHz, DMSO-d_6_) of (*E*)-2-(5-fluoro-1-((1-(2-(2-methyl-5-nitro-1*H*-imidazol-1-yl)ethyl)-1*H*-1,2,3-triazol-4-yl)methyl)-2-oxoindolin-3-ylidene)hydrazine-1-carbothioamide (10b):**


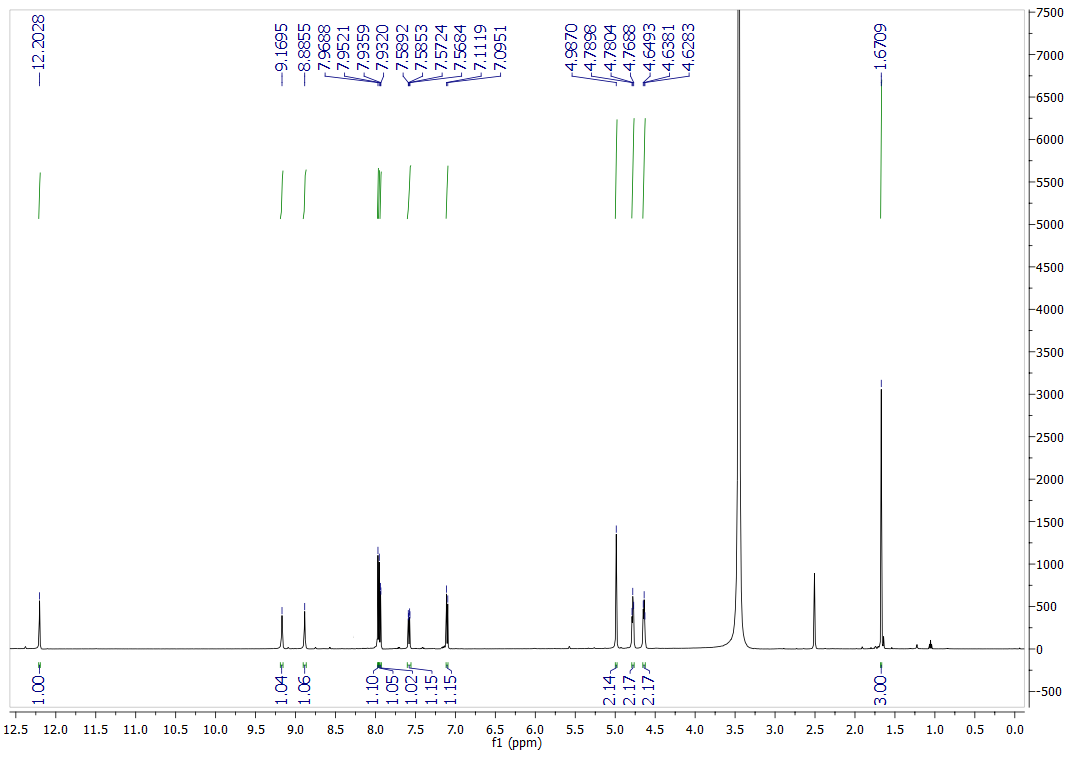


**^13^C NMR (125 MHz, DMSO-*d_6_*) of (*E*)-2-(5-fluoro-1-((1-(2-(2-methyl-5-nitro-1*H*-imidazol-1-yl)ethyl)-1*H*-1,2,3-triazol-4-yl)methyl)-2-oxoindolin-3-ylidene)hydrazine-1-carbothioamide(10b):**


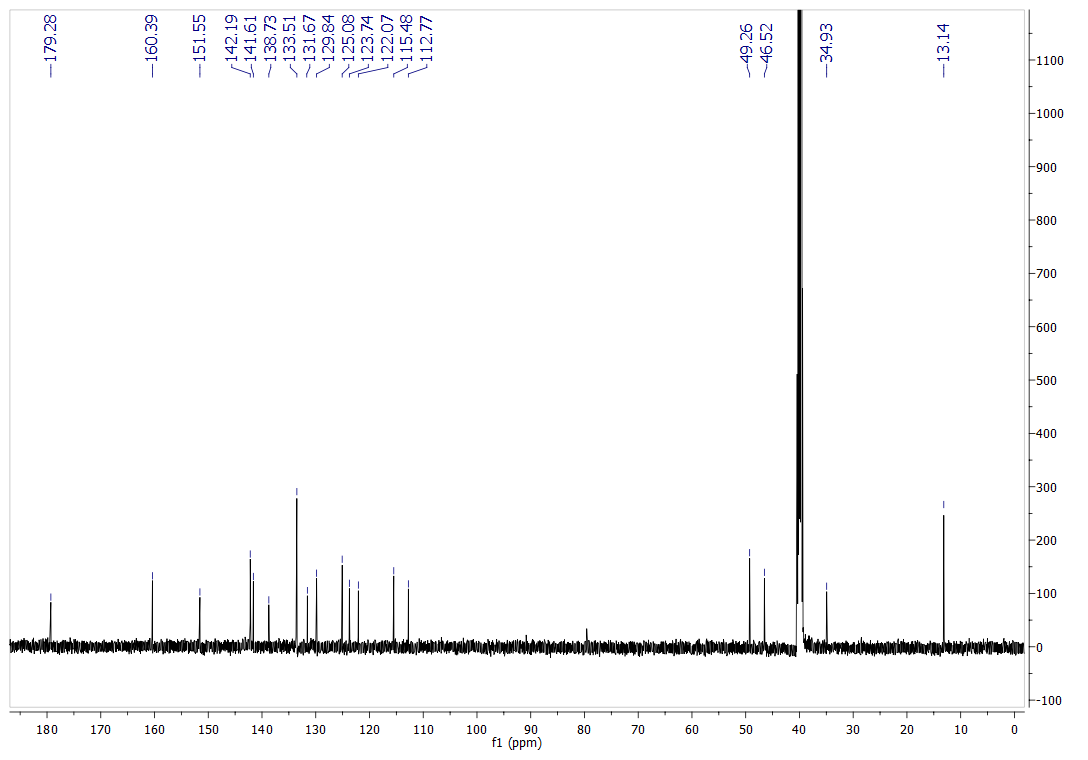


**^1^H-NMR (500 MHz, DMSO-*d_6_*) of 5-fluoro-1-((1-(2-(2-methyl-5-nitro-1*H*-imidazol-1-yl)ethyl)-1*H*-1,2,3-triazol-4yl)methyl)indoline-2,3-dione (8b):**


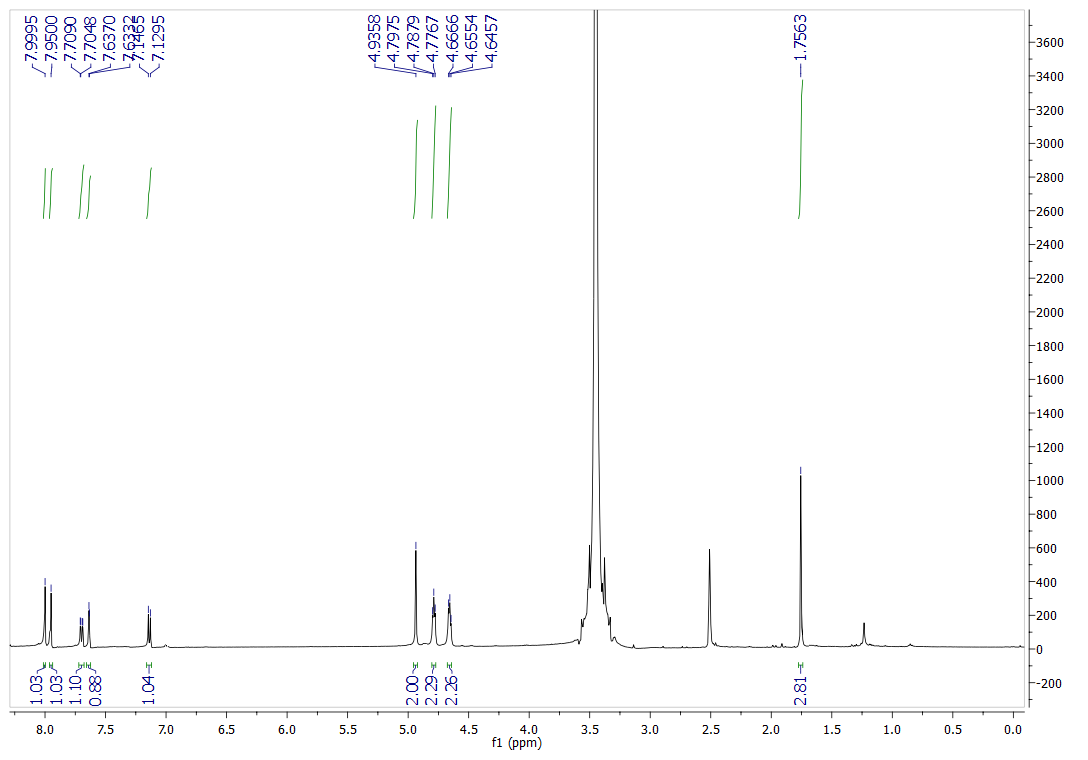


**^13^C-NMR (100 MHz, DMSO-*d_6_*) of 5-fluoro-1-((1-(2-(2-methyl-5-nitro-1*H*-imidazol-1-yl)ethyl)-1*H*-1,2,3-triazol-4yl)methyl)indoline-2,3-dione (8b):**


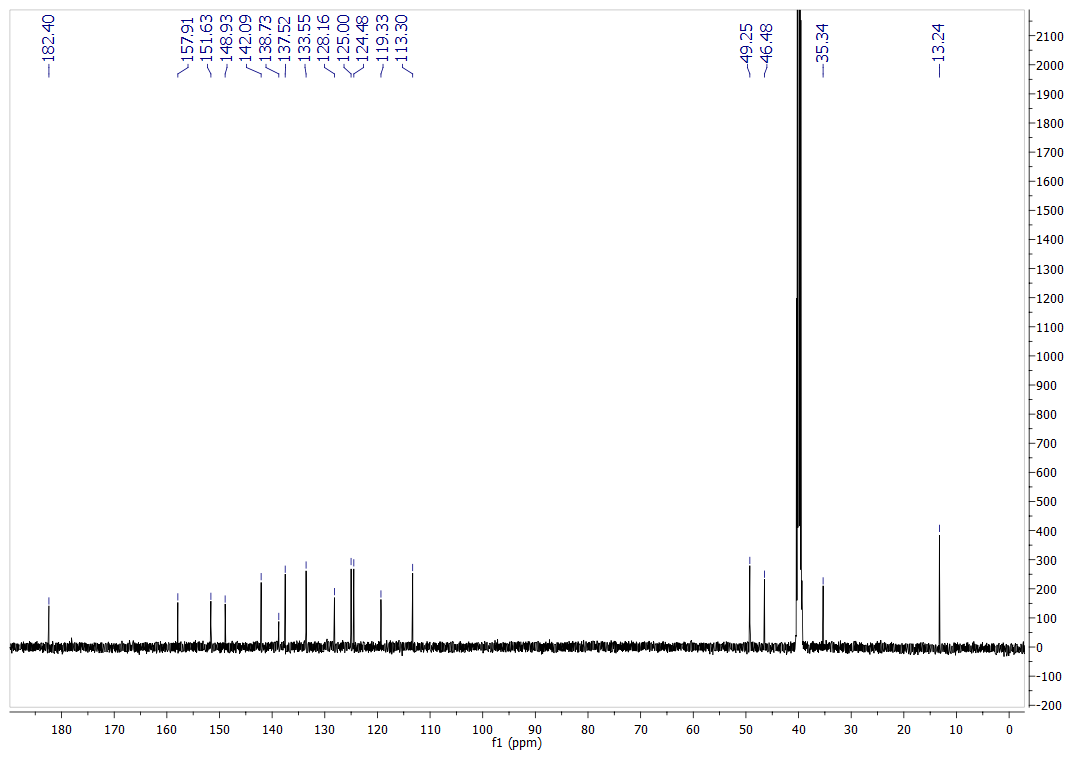


**^13^C Dept-NMR (100 MHz, DMSO-*d_6_*) of 5-fluoro-1-((1-(2-(2-methyl-5-nitro-1*H*-imidazol-1-yl)ethyl)-1*H*-1,2,3-triazol-4yl)methyl)indoline-2,3-dione (8b):**


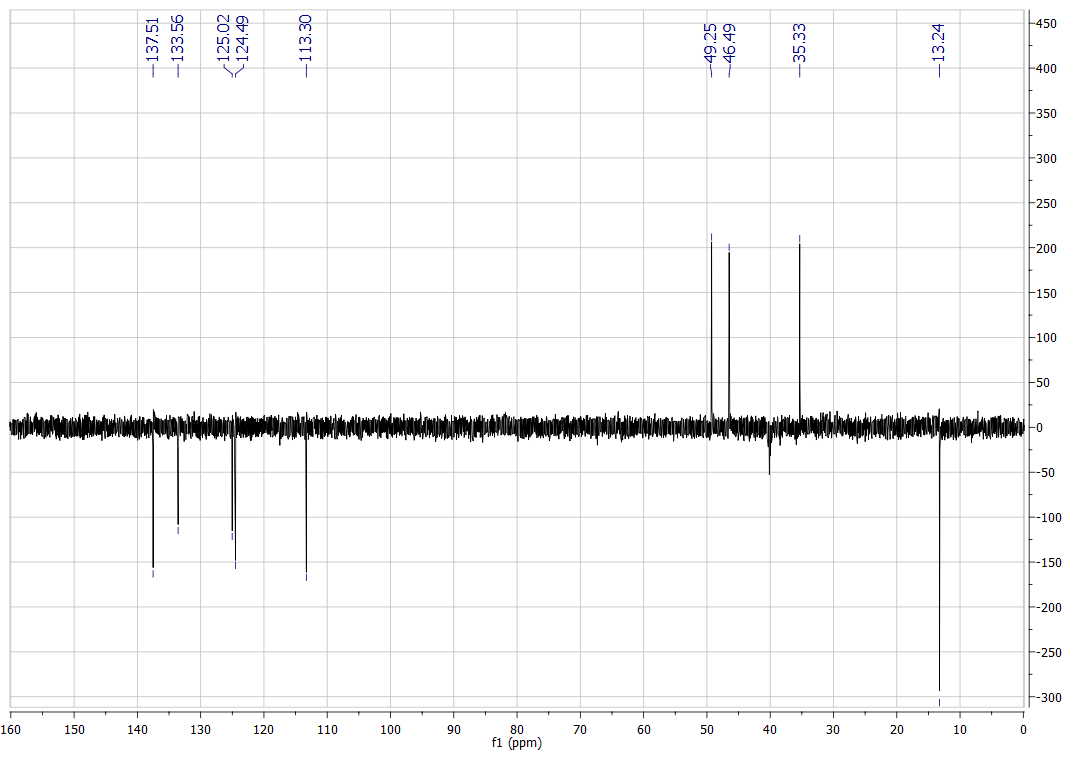


**^1^H NMR (400 MHz, DMSO-*d_6_*) (*E*)-2-(1-((1-(2-(2-methyl-5-nitro-1*H*-imidazol-1-yl)ethyl)-1*H*-1,2,3-triazol-4-yl)methyl)-2-oxoindolin-3-ylidene)hydrazine-1-carbothioamide (10a):**


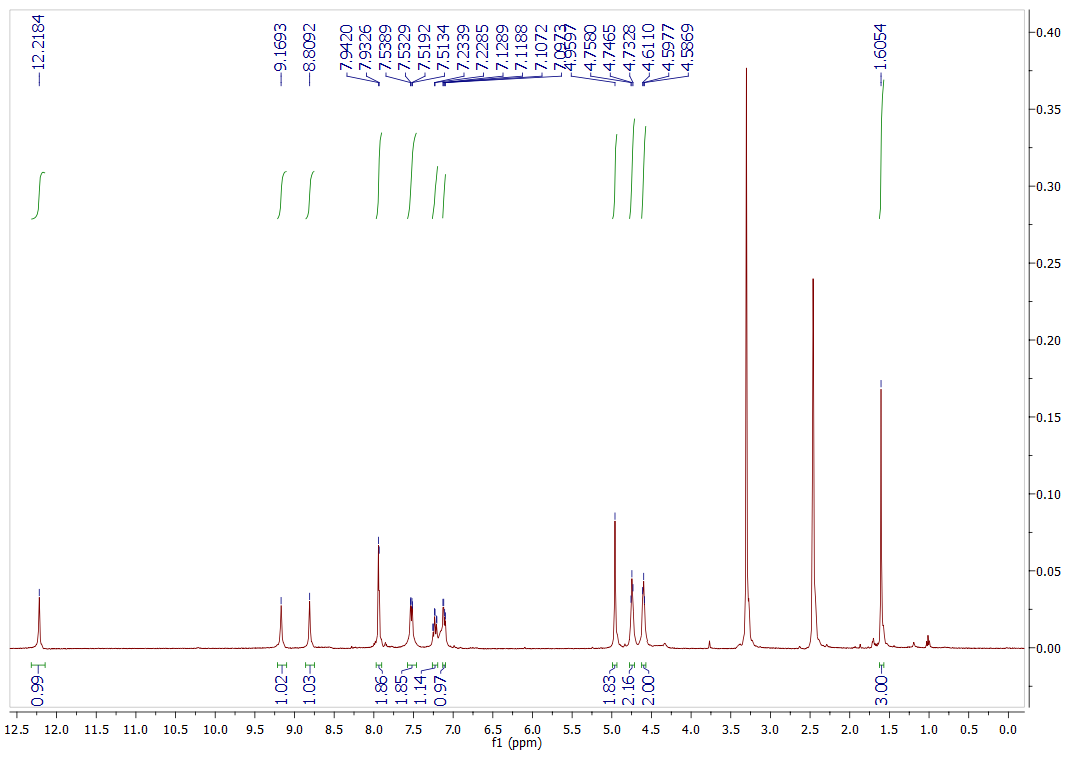


**^1^H NMR (400 MHz, DMSO-*d_6_*+D_2_O) (*E*)-2-(1-((1-(2-(2-methyl-5-nitro-1*H*-imidazol-1-yl)ethyl)-1*H*-1,2,3-triazol-4-yl)methyl)-2-oxoindolin-3-ylidene)hydrazine-1-carbothioamide (10a)**

^
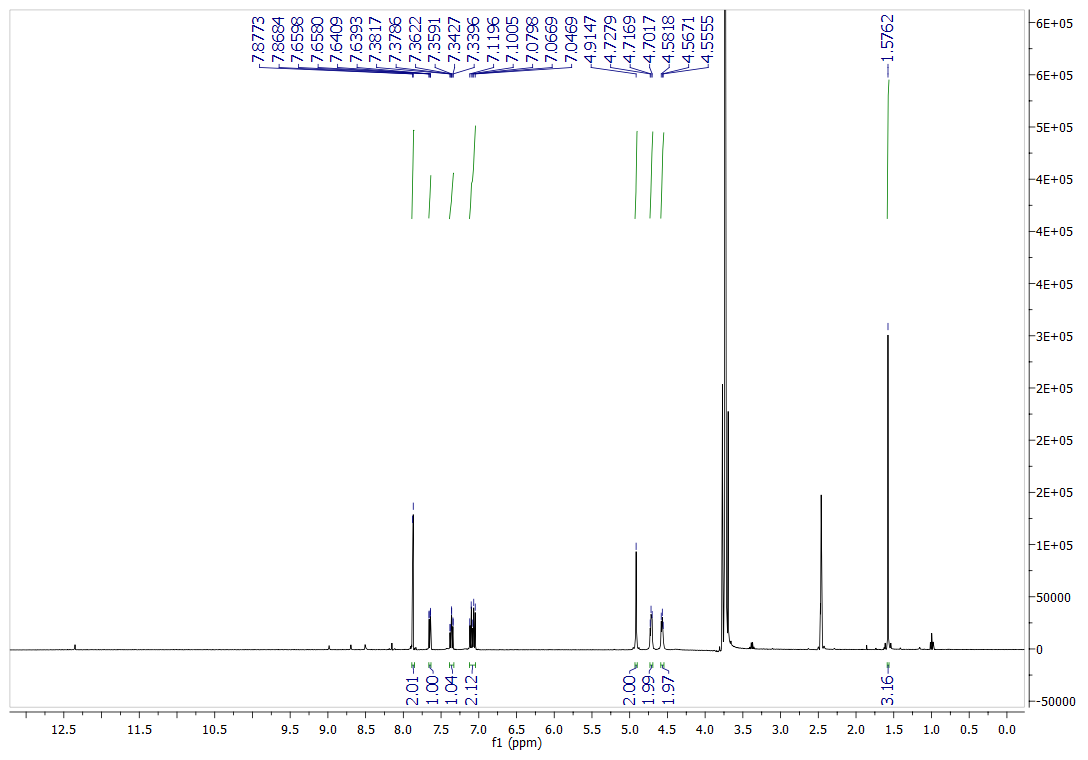
^

**^13^C NMR (100 MHz, DMSO-*d_6_*) of (*E*)-2-(1-((1-(2-(2-methyl-5-nitro-1*H*-imidazol-1-yl)ethyl)-1*H*-1,2,3-triazol-4-yl)methyl)-2-oxoindolin-3-ylidene)hydrazine-1-carbothioamide (10a)**


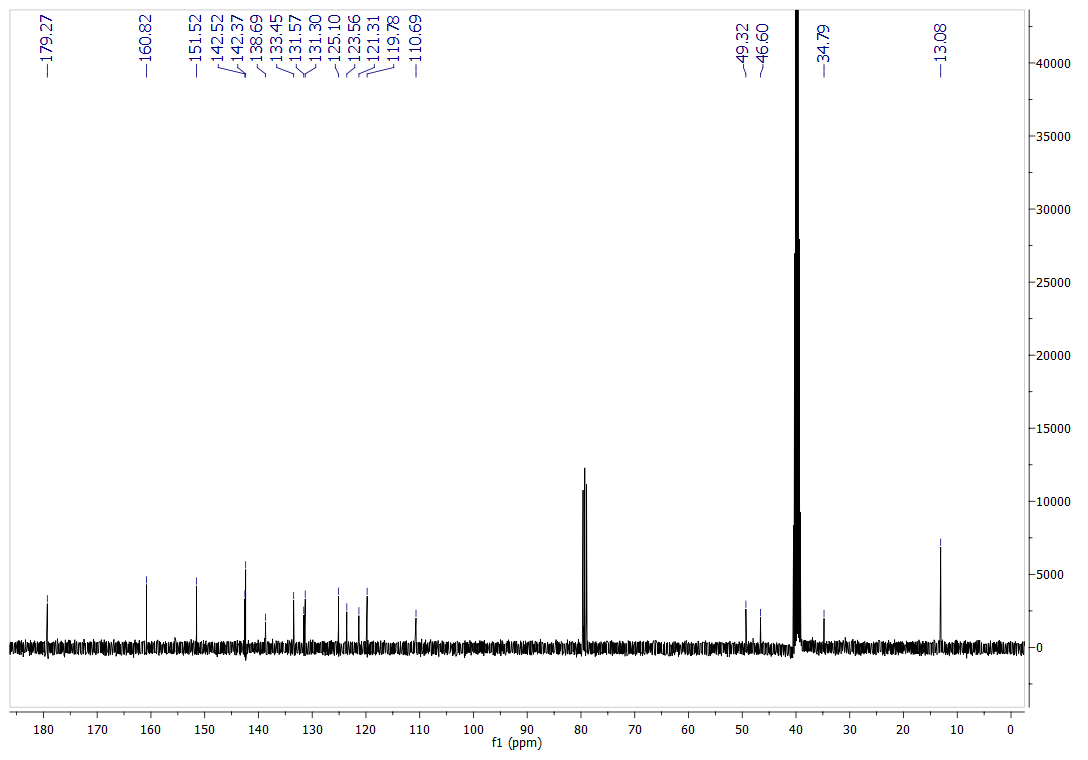


**^1^H NMR (500 MHz, DMSO-*d_6_*) of 1-((1-(2-(2-methyl-5-nitro-1*H*-imidazol-1-yl)ethyl)-1*H*-1,2,3-triazol-4yl)methyl)indoline-2,3-dione (8a):**


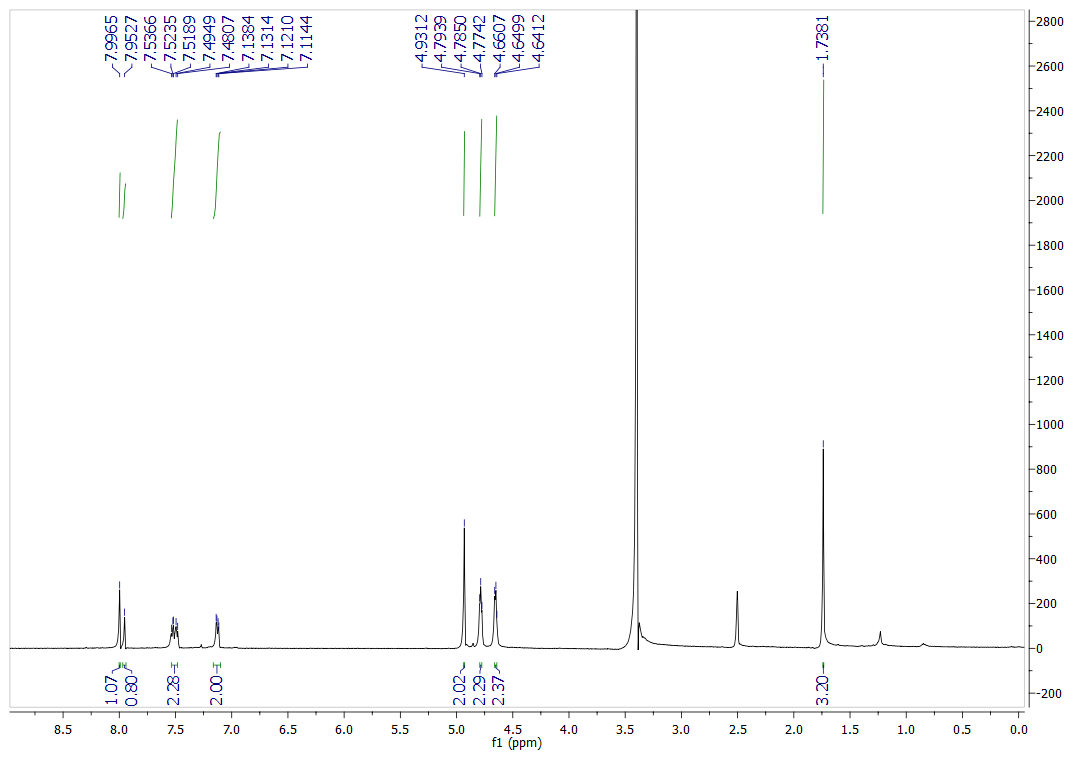


**^13^C NMR (100 MHz, DMSO-*d_6_*) of 1-((1-(2-(2-methyl-5-nitro-1*H*-imidazol-1-yl)ethyl)-1*H*-1,2,3-triazol-4yl)methyl)indoline-2,3-dione (8a):**


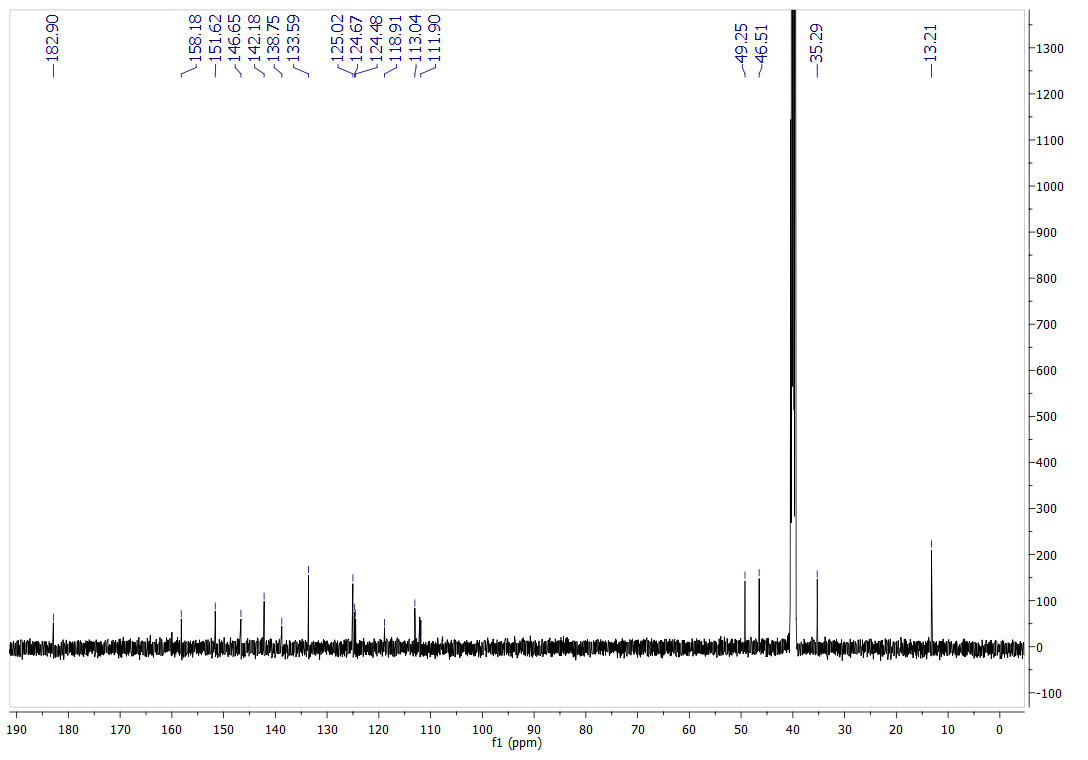

Supplement: Supplementary Data Sheet 1 — 1H, 13C NMR data of all the synthesized compounds along with scanned 1H, 13C and 13C (DEPT) NMR of 8a, 8b, 8c, 9c, 10a, 10b. [file Data_Sheet_1.DOCX]
